# Peak Exposures in Epidemiologic Studies and Cancer Risks: Considerations for Regulatory Risk Assessment

**DOI:** 10.1111/risa.13294

**Published:** 2019-03-29

**Authors:** Harvey Checkoway, Peter S. J. Lees, Linda D. Dell, P. Robinan Gentry, Kenneth A. Mundt

**Affiliations:** ^1^ Department of Family Medicine & Public Health San Diego School of Medicine, University of California La Jolla CA USA; ^2^ Johns Hopkins Bloomberg School of Public Health Baltimore MD USA; ^3^ Ramboll, Amherst MA (LDD) and Monroe LA USA; ^4^ Cardno Chemrisk Boston MA USA

**Keywords:** Cancer epidemiology, peak exposure, risk assessment

## Abstract

We review approaches for characterizing “peak” exposures in epidemiologic studies and methods for incorporating peak exposure metrics in dose–response assessments that contribute to risk assessment. The focus was on potential etiologic relations between environmental chemical exposures and cancer risks. We searched the epidemiologic literature on environmental chemicals classified as carcinogens in which cancer risks were described in relation to “peak” exposures. These articles were evaluated to identify some of the challenges associated with defining and describing cancer risks in relation to peak exposures. We found that definitions of peak exposure varied considerably across studies. Of nine chemical agents included in our review of peak exposure, six had epidemiologic data used by the U.S. Environmental Protection Agency (US EPA) in dose–response assessments to derive inhalation unit risk values. These were benzene, formaldehyde, styrene, trichloroethylene, acrylonitrile, and ethylene oxide. All derived unit risks relied on cumulative exposure for dose–response estimation and none, to our knowledge, considered peak exposure metrics. This is not surprising, given the historical linear no‐threshold default model (generally based on cumulative exposure) used in regulatory risk assessments. With newly proposed US EPA rule language, fuller consideration of alternative exposure and dose–response metrics will be supported. “Peak” exposure has not been consistently defined and rarely has been evaluated in epidemiologic studies of cancer risks. We recommend developing uniform definitions of “peak” exposure to facilitate fuller evaluation of dose response for environmental chemicals and cancer risks, especially where mechanistic understanding indicates that the dose response is unlikely linear and that short‐term high‐intensity exposures increase risk.

## 1. INTRODUCTION

Regulatory risk assessment is a process that focuses on identifying potentially adverse health effects associated with agents of concern, and for each adverse effect, characterizing a dose–response function and unit risk numbers. Toxicology studies that can characterize biological uptake and pathogenesis mechanisms are important components of hazard identification and risk assessment. Epidemiologic studies, especially those of well‐characterized occupational groups, often identify associations between exposures and increased disease risk. Depending on available detail and chemical specificity of exposure assessment, dose–response estimation may be based on quantitative (usually cumulative) or qualitative (yes/no or high/medium/low) relative exposure estimates. In some instances, cruder surrogates of exposure are based only on overall duration of employment or duration of employment in jobs classified by exposure potential, such as “presumed exposed/unexposed.” When quantitative exposure data of sufficient validity and specificity are available, epidemiologic studies are preferred over animal studies for quantitative dose–response assessments that can be applied to derive relative toxicity values, such as inhalation unit risk (IUR) values. Consequently, scientifically rigorous investigations of dose–response relations between exposures to environmental chemicals and cancer risks are dependent on valid quantitative characterization of exposure.

In conducting quantitative risk assessments, individual‐level exposure estimates are required, even if derived using ecological exposure assessment (i.e., group level, such as a similar exposure group in occupational studies). However, several different quantitative exposure metrics can be derived, and those that most accurately reflect underlying biological mechanisms should more accurately reflect the true underlying dose–response gradient. Typically, in epidemiologic research, actual doses of chemical compounds delivered to a target tissue rarely can be determined. A notable exception is the measurement of valid biomarkers of exposure, some of which reflect recent exposures (e.g., urinary chromium), whereas others reflect long‐term (e.g., polychlorinated biphenyls or PCBs in blood sera) or even cumulative (e.g., tibial lead) exposure. Given that many cancers known or presumed to be caused by environmental agents have long latency periods, epidemiologists can improve individual‐level exposure estimates by considering time windows of relevant exposure. In theory, estimating time‐dependent exposure responses for various exposure metrics would lead to more sophisticated characterizations of risk, and therefore more valid risk assessments for policy and regulatory decision making.

For some carcinogens, disease processes conceivably depend on the dose rate and the concentration of the chemical (or its metabolites) that reach the target tissue, possibly overwhelming metabolic pathways that can eliminate or detoxify lower concentrations. In this case, an appropriate exposure metric might be the maximum concentration encountered (i.e., “peak” exposure), or the amount of time exposure exceeds some threshold. The identification of peak exposures (or simply the exceedance of a critical threshold) is common in evaluating acute effects; however, for nearly all cancers and other noninfectious diseases that require considerable time to elapse between exposure and disease detection, characterizing the role of historical peak exposures remains challenging.

In this review, our primary objective is to consider “peak exposure” as variously defined in the context of epidemiologic studies of recognized carcinogens and related epidemiology‐based cancer risk assessments. We limit our review to occupational rather than general nonworkplace environmental exposures (e.g., outdoor air pollution) because the workplace often represents the “high dose” scenario. Occupational exposure measures are frequently more detailed and quantitative than exposures that occur in the general environment. Moreover, occupational epidemiologic research findings often have important risk assessment and policy implications for ambient environmental exposures. Although some low‐concentration exposures likely contribute to disease risk, we focus here on ways that occupational epidemiology might improve understanding of cancer risks associated with brief or intermittent exposures to high concentrations that may be characterized as peak exposure.

Our research question was initially motivated by a practical issue raised by the U.S. National Research Council (NRC, 2011) regarding the hazard characterization of formaldehyde as potentially carcinogenic to humans (Baan et al., 2009; International Agency for Research on Cancer [IARC], 2006). While the epidemiologic evidence suggested some association between risks for nasopharyngeal cancer (NPC) and myeloid leukemia and peak formaldehyde exposure metrics, there remains limited and inconsistent evidence for increased risks of these cancers associated with cumulative exposure, the conventional dose metric. Nevertheless, the U.S. Environmental Protection Agency (US EPA, 2010) draft assessment of formaldehyde was based on the cumulative exposure metric from selected relevant occupational epidemiologic studies (Beane Freeman et al., 2009; Hauptmann, Lubin, Stewart, Hayes, & Blair, 2003), and not peak exposure, to derive an IUR (US EPA, 2010). In 2018, US EPA proposed regulation “designed to increase transparency of the assumptions underlying dose–response models” given “growing empirical evidence of nonlinearity in the concentration‐response function” for environmental exposures and health effects including cancers. The proposed rule cautions that “EPA should also incorporate the concept of model uncertainty when needed as a default to optimize low dose risk estimation based on major competing models, including linear, threshold, and U‐shaped, J‐shaped, and bell‐shaped models” [40 CFR Part 30 RIN 2080‐AA14]. Promulgation of this rule language likely will revive interest in understanding alternative exposure metrics as well, including peak exposure.

## 2. METHODS

We conducted a literature search in PubMed (including MEDLINE, 1966–present) to identify epidemiologic studies in which results included estimates of relative risks in relation to some form of peak chemical exposure metric. Initial search terms included “peak,” “exposure,” and “epidemiology.” We screened titles and abstracts from this initial search and then refined the search to include terms for chemicals identified in the abstracts: acrylonitrile, benzene, 1,3‐butadiene (BD), ethylene oxide (EO), formaldehyde, methylene chloride, styrene, trichloroethylene (TCE), and tetrachlorodibenzo‐p‐dioxin (TCDD).

We separately reviewed the IARC Monographs website^1^ to identify chemical agents characterized as having “sufficient evidence in humans” or “limited evidence in humans” of causing lymphohematopoietic malignancies (LHMs) based on epidemiologic studies. Our focus on LHMs was based on three factors: (1) knowledge of cancer hazard characterization driven by epidemiologic studies reporting increased myeloid leukemia associated with peak formaldehyde exposure categories (Beane Freeman et al., 2009; Hauptmann et al., 2009); (2) the assumption that LHMs have shorter disease induction and latency intervals (i.e., 2 to <10 years) than solid tumors (i.e., ≥15 or ≥20 years) and/or may be more sensitive or responsive to “peak” exposures as demonstrated by induction of leukemias among individuals administered high doses of chemotherapeutic agents (IARC, 2012a; US EPA, 2012); and (3) our assumption that LHM risks associated with peak exposures should be more easily identified over shorter follow‐up periods in cohort studies, which admittedly is speculative, but helpful in providing a focused literature review in which peak exposure concepts and analytic methods can be explored.

Next, we reviewed the IARC Monographs for each of the identified chemical agents to determine which epidemiologic studies were relied upon for the cancer hazard characterization, and if any provided information about peak exposures. We separately reviewed the US EPA Integrated Risk Information System (IRIS) database to identify which epidemiologic studies, if any, were relied upon for dose–response assessment, and which of these studies also reported risks in relation to peak exposures.^2^

For any epidemiologic study that reported peak exposure information, we extracted information on the exposure reconstruction approach, choice of exposure modeling approach, definitions of peak exposure, factors considered as part of the time dependency of cancer (e.g., estimation of cancer diagnosis latency), risk estimates in relation to peak exposure metrics, whether sensitivity analyses were conducted to assess exposure misclassification (and results of the analysis, if any), and considerations or discussion of validity and reliability of the exposure assessment.

## 3. CASE STUDIES

For LHMs, IARC Working Groups have characterized the epidemiologic evidence as “sufficient” for benzene (acute myeloid leukemia [AML]/acute nonlymphocytic leukemia [ANLL]), formaldehyde (leukemia), and BD (all LHMs). The epidemiologic evidence has been characterized as “limited” for dichloromethane (methylene chloride) (non‐Hodgkin lymphoma [NHL]), ethylene oxide (NHL, multiple myeloma, chronic lymphocytic leukemia), styrene (LHMs), and TCE (NHL). Each of these has been classified as Group 1 (carcinogenic to humans) by IARC, except for methylene chloride and styrene, which are classified as Group 2A (probably carcinogenic). The IARC also has identified sufficient evidence in humans of NRC for formaldehyde, and kidney cancer for TCE (as well as “limited” evidence in humans for liver cancer). The IARC also reported limited evidence in humans of breast cancer for EO, and EO was upgraded to a Group 1 carcinogen based on mechanistic data and sufficient evidence in experimental animals although the epidemiological evidence does not demonstrate an increased risk of breast cancers.

In addition to these substances, our literature search also identified acrylonitrile and TCDD as chemicals for which peak exposure was evaluated in epidemiologic studies, and which IARC has classified as possibly carcinogenic to humans (Group 2B) and carcinogenic to humans (Group 1), respectively. After reviewing full text articles, we included acrylonitrile in our review. We excluded TCDD from further consideration because peak TCDD exposures were reported as the result of an industrial accident in Seveso, Italy (Bertazzi, Bernucci, Brambilla, Consonni, & Pesatori, 1998) and thus were unusual and not representative of peak exposures that describe the higher exposure concentrations associated with day‐to‐day occupational exposure variability.

For each identified substance, Supporting Information Table SI summarizes its common uses, existing occupational exposure limits, the IARC characterization as human carcinogens, the US EPA IURs for carcinogenicity (i.e., the upper‐bound excess lifetime cancer risk estimate to result from continuous exposure at a concentration of 1 μg/m^3^), and references for epidemiologic studies with quantitative or semiquantitative exposure metrics that informed the characterization of the human cancer hazard by IARC or the dose–response assessment by the US EPA. Supporting Information Table SII describes the rationale for evaluating peak exposures in relation to risk for each reference in which a peak exposure metric was used (where available).

### 3.1. Benzene and LHM

The US EPA (1998) relied upon epidemiologic studies (Rinsky et al., 1987; Rinsky, Young, & Smith, 1981) for dose–response assessment using cumulative exposure to derive an IUR for benzene (Supporting Information Table SIM).

Collins, Ireland, Buckley, and Shepperly (2003) evaluated associations between peak exposure estimates and risk of LHMs in a study of workers at a U.S. benzene‐derived chemicals manufacturing plant in a pooled study of petroleum industry workers in Canada, the United Kingdom, and Australia (Glass et al., 2010; Glass, Schnatter, Tang, Irons, & Rushton, 2014; Rushton, Schnatter, Tang, & Glass, 2014; Schnatter, Glass, Tang, Irons & Rushton, 2012) and a study of Norwegian offshore oil drillers (Stenehjem et al., 2015) (Table I). Peaks in these studies were defined, respectively, as number of days with 15‐minute excursions >100 parts per million (ppm) (Collins et al., 2003), at least one year in job with >3 ppm for 15–60 minutes at least weekly (yes/no) (Glass et al., 2010, 2014; Rushton et al., 2014; Schnatter et al., 2012), or a semiquantitative score (STEL score) that reflected the frequency (often/sometimes/never) of exceeding the Norwegian short‐term exposure limit (STEL) of 3 ppm for 15 minutes (Stenehjem et al., 2015).

**Table I:** Benzene Exposure and Lymphohematopoetic Malignancies (LHM)

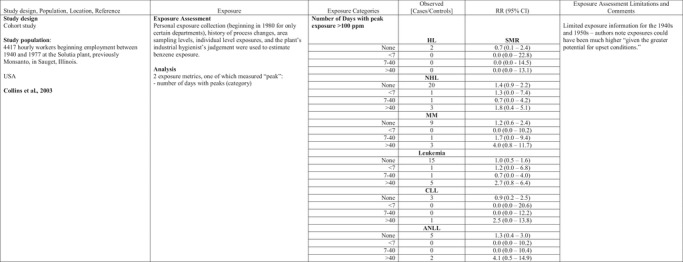
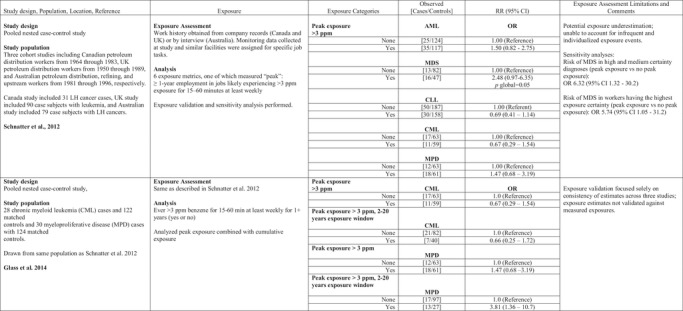
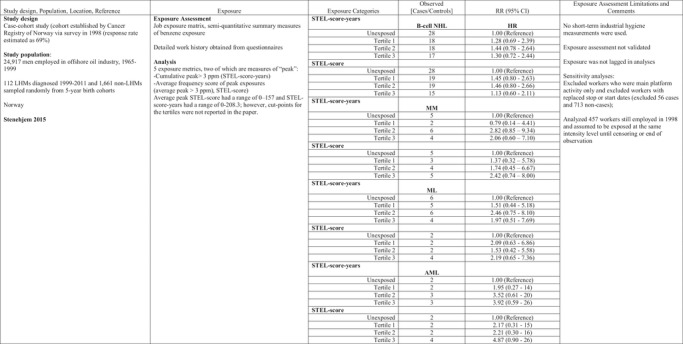

AML, acute myeloid leukemia; ANLL, acute non‐lymphocytic leukemia; CLL, chronic lympocytic leuekmia; CML, chronic myeloid leukemia; HL, Hodgkin lymphomas; HR, hazard ratio; MDS, myelodysplastic syndrome; MM, multiple myeloma; MPD, myeloproliferative disorder; NHL, non‐Hodgkin lymphoma; OR, odds ratio; RR, relative risk; STEL, short‐term exposure limit.

Risks for specific LHMs were not consistently increased in relation to any of the peak exposure metrics in these studies (Table I). Schnatter et al. (2012) reported increased risk of myelodysplastic syndrome in workers exposed to peaks >3 ppm (Table I) and in relation to cumulative exposure: OR 1.73 (95% CI 0.55–5.47) for 0.348–2.93 ppm‐years and OR 4.33 (95% CI 1.31–14.3) for >2.93 ppm‐years (compared to the referent group exposed to ≤0.348 ppm‐years).

Stenehjem et al. (2015) reported risk of AML increased with cumulative exposure (trend test *p* = 0.052) more strongly than with cumulative peak exposure (trend test *p* = 0.166) or with average peak exposure (trend test *p* = 0.056). Cumulative peak exposure was calculated by multiplying the STEL score by the duration of employment for each of the jobs and summing over all jobs while average peak exposure was calculated by dividing cumulative peak exposure by exposure duration. Similarly, multiple myeloma increased with cumulative exposure (trend test *p* = 0.024) and average peak exposure, but not with cumulative peak exposure. The trend test was not significant for either peak exposure metric. Exposure was not lagged, and most workers were exposed to benzene for fewer than 15 years.

Collins et al. (2003) reported no increased risks for all leukemias combined or ANLL associated with cumulative exposure, and no trends by peak exposure for any of several LHMs; however, the highest standardized mortality ratios (SMRs) for multiple myeloma and ANLL were seen in the highest peak exposure category (>40 days with peak exposure >100 ppm), although based on very few (3 and 2, respectively) observed deaths.

### 3.2. Formaldehyde and NPC and LHM

IARC Working Groups have classified formaldehyde as carcinogenic to humans, causing NPC (IARC, 2006, 2012b) and leukemia, in particular myeloid leukemia (IARC, 2012b), based on results generated when exposure was characterized as a “peak exposure.” The US EPA Draft IRIS assessment, however, used cumulative exposure metrics from epidemiologic studies (Beane Freeman et al., 2009; Hauptmann et al., 2003) for the dose–response assessment to derive an IUR value for the combined risks of leukemia, Hodgkin lymphoma, and NPC (Supporting Information Table SI).

Peak formaldehyde exposures were considered in two studies initiated by the U.S. National Cancer Institute, one of workers from multiple industries with formaldehyde exposure (e.g., formaldehyde resin manufacturing) and the other on funeral directors and embalmers. In the U.S. formaldehyde producers and users study (Beane Freeman et al., 2009, 2013), peak exposure was defined as short‐term exposures (generally less than 15 minutes) that exceeded job‐specific 8‐hour time‐weighted average (TWA) exposure estimates (Stewart et al., 1986). For workers classified as not having held jobs with exposures exceeding those 8‐hour TWA levels, peak exposures were defined as the job‐specific 8‐hour TWA. Beane Freeman et al. (2009) reported associations with peak exposure (Table II), but not for cumulative exposure, average intensity, duration of exposure, or cumulative number of peaks for all LHM and for ML. In an independent reanalysis of the data, peaks were redefined as absolute peaks, ≥1 continuous month of employment in jobs identified in the original exposure characterization as likely having short‐term exposure excursions of ≥2 ppm to <4 ppm or ≥4 ppm on a weekly or daily basis for all workers in the cohort (Checkoway et al., 2015). Using an absolute peak metric, Checkoway et al. (2015) reported an attenuated relation for ML and no association for AML, as only four of the 34 workers with AML as the cause of death had been classified as having peak exposures in the 20 years preceding their deaths. Separately, Beane Freeman et al. (2013) reported increased lung cancer mortality (SMR 1.15; 95% CI 1.07–1.20) and excess NPC mortality (SMR 1.84, 95% CI 0.84–3.49) for workers assigned to their highest peak exposure category; internal analyses by peak exposure, however, generated no positive associations with lung cancer, and consistently increased mortality from NPC for the highest category of each exposure metric: peak exposure ≥4 ppm (RR 7.66, 95% CI 0.94–62.3), average intensity ≥1 ppm (RR 11.54, 95% CI 1.38–96.8), and cumulative exposure ≥5.5 ppm (RR 2.94, 95% CI 0.65–13.3).

**Table II:** Formaldehyde Peak Exposure and LHMs

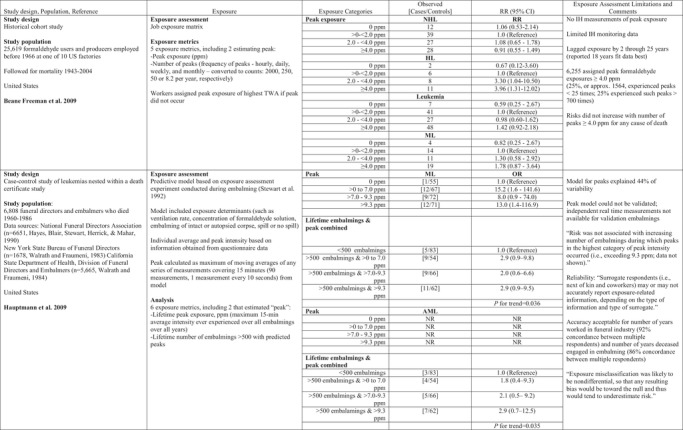

AML, acute myeloid leukemia; HL, Hodgkin lymphoma; ML, myeloid leukemia; NHL, non‐Hodgkin lymphoma; ppm, parts per million; NR, not reported; RR, relative risk; TWA, time‐weighted average.

In the funeral industry workers study, lifetime peak formaldehyde exposure was defined as the maximum 15‐minute average exposure intensity estimate derived over the entire duration of employment (Hauptmann et al., 2009). A predictive model was developed based on a literature review and walk‐through surveys at funeral homes, resulting in three determinants of exposure: ventilation (low, moderate, high) in the embalming room, concentration of formaldehyde in the embalming solution (high vs. low solution strength), and whether the embalming was performed on an intact or autopsied body (Hornung et al., 1996). The investigators conducted limited sampling of formaldehyde concentrations in the breathing zone of the embalmer and three locations in the room, based on the combinations of the determinants of exposure. The model defined peak exposure as the maximum of moving averages of any series of measurements covering 15 minutes (90 measurements, i.e., one measurement every 10 seconds). In addition, the investigators calculated the lifetime number of embalmings that were associated with predicted peaks exceeding a certain level. For the evaluation of the average exposure model, the investigators compared model predictions to measurements from 15 independent embalmings and reported that the model overestimated measured (average, not peak) formaldehyde exposure by 35%. The authors reported that the peak model could not be validated due to lack of real‐time measurements. The authors described increasing risks of AML in relation to peak exposure only in a model that included embalmers and directors who had performed 500 or more embalmings (Table II).

Two other large industrial cohort studies examined LHM risks associated with formaldehyde exposure; however, neither study used peak exposure or any reasonable surrogate. The NIOSH garment workers study reported a geometric mean concentration of 0.15 ppm measured from the 1980s and reported that exposure levels at the three facilities were likely to have been similar to formaldehyde exposures experienced in the NCI and U.K. cohorts in earlier years (Meyers, Pinkerton & Hein, 2013). This conclusion was based on average concentrations of formaldehyde at eight garment factories in 1966 (range of average concentrations 0.3–2.7 ppm), and measured formaldehyde concentrations in cutting rooms reported to be as high as 10 ppm in 1968, and subsequently reduced to 2 ppm in 1973 (as reported in IARC, 2006). The U.K. cohort study of formaldehyde producers and users reported that the high‐exposure category represented average concentrations of 2 ppm or higher (Coggon, Ntani, Harris, & Palmer, 2014). Overall, workers experienced higher average exposures in the United Kingdom compared to the United States: 3,991 (28%) of the U.K. workers were assigned to average intensity identified as “high” (i.e., ≥2 ppm) and 3,927 (15%) of the NCI cohort were reported by Beane Freeman et al. (2009) to be assigned to average intensity ≥1 ppm.

### 3.3. Styrene and/or BD and LHM

Styrene and butadiene are common co‐exposures in the synthetic rubber industry and have been considered together and separately in analyses of cancer risks in a series of studies conducted by University of Alabama Birmingham researchers (Cheng, Sathiakumar, Graff, Matthews & Delzell, 2007; Delzell, Macaluso, Sathiakumar, & Matthews, 2001; Delzell et al., 1996; Graff et al., 2005; Macaluso et al., 1996; Macaluso, Larson, Lynch, Lipton & Delzell, 2004; Sathiakumar, Brill, Leader, & Delzell, 2015). IARC (2012b) has characterized BD as carcinogenic to humans based on limited epidemiologic evidence for a causal association with NHL, multiple myeloma, and chronic lymphocytic leukemia. More recently, IARC characterized styrene as probably carcinogenic to humans based on limited evidence from epidemiologic studies of LHMs and myeloid leukemia, as well as sinonasal adenocarcinoma (Kogevinas et al., 2018). An IUR of 3 × 10^−5^ per μg/m^3^ was derived for butadiene, in part based on these epidemiologic studies; however, an IUR was not derived for styrene (Supporting Information Table SI).

Graff et al. (2005) evaluated relative risk of leukemia mortality in relation to exposure to BD, styrene, and dimethyldithiocarbamate (DMDTC), an immune system depressant. The investigators derived several estimates of exposure for 16,579 workers at synthetic rubber production facilities, including cumulative exposure (ppm‐years for BD and styrene), the annual number of peaks for BD (number of occurrences in which BD exposure exceeded the peak level of 100 ppm), and annual number of peaks for styrene (number of occurrences in which styrene exposure exceeded the peak level of 50 ppm) (Macaluso et al., 2004). In models that included only styrene, relative risks of leukemia mortality were statistically significantly increased for workers in the three highest peak exposure categories after adjusting for age and years since hire (Table III). The relative risk estimates remained increased after adding BD peaks and cumulative exposure to DMDTC to the model (Table III). Separately, the authors reported that cumulative exposure to BD was associated with increased mortality from all leukemias, chronic myelogenous leukemia and chronic lymphocytic leukemia, although mortality risks were attenuated after adjusting for styrene and DMDTC. A positive exposure–response pattern was not seen with either styrene or DMDTC after controlling for BD exposure.

**Table III:** Styrene Peak Exposure and LHMs

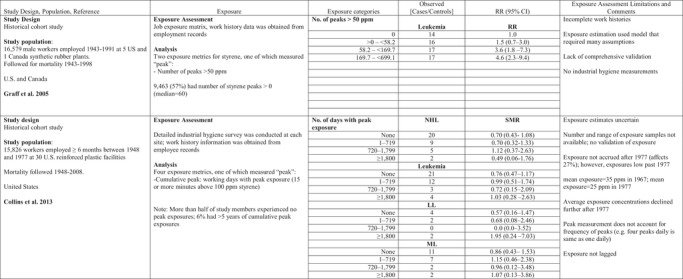

HL, Hodgkin lymphoma; LL, lymphatic leukemia; NHL, non‐Hodgkin lymphoma; ppm, parts per million; ML, myeloid leukemia; RR, relative risk; SMR, standardized mortality ratio.

Styrene exposures are common in the reinforced plastics and composite industry, in which Collins, Bodner, and Bus (2013) examined mortality rates in relation to cumulative exposure, duration of exposure, peak exposures, and average exposure. The peak exposure metric reflected days with 15 or more minutes above 100 ppm styrene (reportedly the lowest level at which irritation from styrene occurs). No patterns of increasing risks of all LHM combined or any specific LHM were reported in relation to days with peak exposure above 100 ppm styrene (Table III). The Danish Cancer Registry linkage study of reinforced plastics industry workers evaluated cumulative exposures to styrene in relation to leukemias and lymphomas (Christensen et al., 2018) and sinonasal carcinoma (Nissen et al., 2018). These studies were identified as the most informative by the IARC working group; however, the studies of the Danish reinforced plastic industry did not report information on risks related to peak exposures or short‐term exposures, despite a substantial number (2,207) of short term (<1 hour) samples collected during 1960–1996 (short‐term exposures as high as 2,720 mg/m^3^) (Kolstad, Sonderskov, & Burstyn, 2005).

### 3.4. TCE and Leukemia, Lymphoma, and Kidney Cancer

IARC has classified TCE as carcinogenic to humans, causing cancer of the kidney (IARC, 2014). The epidemiologic evidence was considered sufficient for a majority of the IARC Working Group, and a minority considered the evidence to be limited. The Working Group also reported limited epidemiologic evidence for NHL (IARC, 2014) and liver cancer in relation to TCE. The US EPA based its dose–response analysis on cumulative exposure from epidemiologic studies (Charbotel, Fevotte, Hours, Martin & Bergeret, 2006; Raaschou‐Nielsen et al., 2003) and derived an IUR for combined risks from kidney cancer, liver cancer, and NHL (US EPA, 2011b).

In addition to categorical cumulative exposure assignments (1–150 ppm‐years, 155–335 ppm‐years, and >335 ppm‐years), Charbotel et al. (2006) also evaluated peak exposure in a case‐control study of 86 renal cancer cases and 316 hospital and clinic‐based controls in France. Peak exposure was not clearly defined but peak effects were evaluated in combination with cumulative exposure by assigning exposed cases and controls to one of four exposure categories: exposed to low or medium cumulative dose without peaks, low or medium cumulative dose with peaks, high cumulative dose without peaks, and high cumulative dose with peaks. After adjusting for smoking and BMI, Charbotel et al. (2006) reported a significantly increased odds ratio for renal cell carcinoma for high cumulative dose without peaks and high cumulative dose with peaks (Table IV).

**Table IV:** Trichloroethylene and Cancers

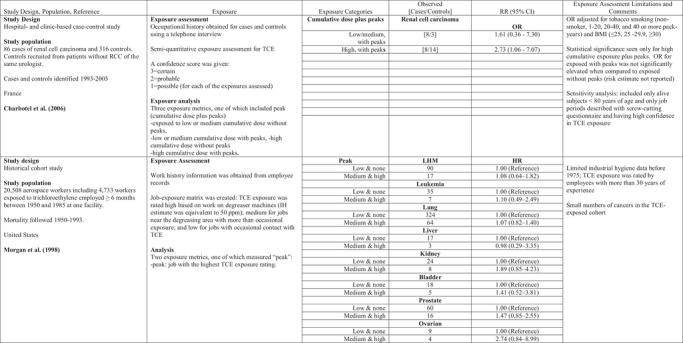
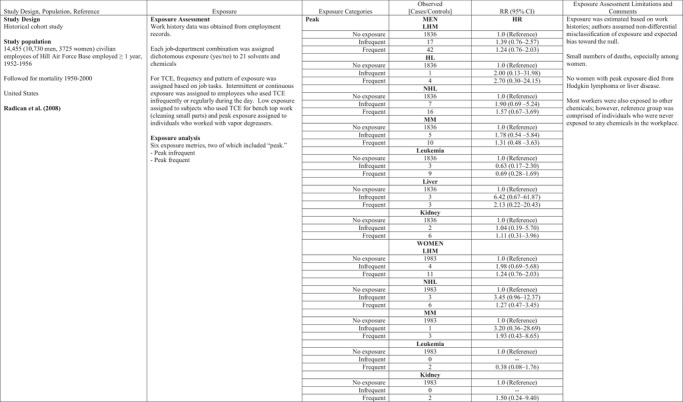

HL, Hodgkin lymphoma; HR, hazard ratio; MM, multiple myeloma; NHL, non‐Hodgkin lymphoma; ppm, parts per million; OR, odds ratio.

Two epidemiologic cohort studies of aircraft workers examined peak exposure to TCE. Morgan, Kelsh, Zhao and Heringer (1998) studied 20,508 workers employed at least six months between 1950 and 1985 at an aircraft manufacturing facility in Arizona. The cohort was followed for mortality from 1950 to 1992. A total of 4,733 workers were classified as exposed to TCE during their work. Peak exposures were defined as the job with the highest TCE exposure rating. Peak exposures were analyzed by comparing workers with high and medium TCE exposure jobs (where peak exposures were assumed to have occurred) to workers with no and low TCE exposure. High TCE exposures, estimated to be above 50 ppm, involved assignments to degreaser machines, while work in areas near the vapor degreasing unit and with more than occasional contact with TCE were assigned medium TCE exposure. No statistically significant increased risks of leukemia, lymphoma, or kidney cancer were observed for workers with peak exposures when compared to workers with low (occasional contact with TCE) and no TCE exposure (Table IV).

Radican, Blair, Stewart, and Wartenberg (2008) followed mortality from 1953 through 2000 in a cohort of 14,455 aircraft maintenance workers employed for one year or more between 1952 and 1956 as civilians at Hill Air Force Base. Workers were assigned to categories of TCE exposure based on job tasks that identified four patterns of frequency and exposure: intermittent exposure (i.e., used TCE infrequently during the day), continuous exposure (i.e., used TCE regularly throughout the day), low exposure (i.e., used TCE for bench top work to clean small parts), or peak exposure (i.e., workers who used vapor degreasers). Each worker was assigned to one of four categories of exposure: low intermittent exposure, low continuous exposure, infrequent peak exposure, and frequent peak exposure. This exposure metric differed somewhat from an earlier report on the same cohort (Blair, Hartge, Stewart, McAdams, & Lubin, 1998) that described results according to three categories of TCE exposure: low‐level intermittent exposure, low‐level continuous exposure, and frequent peaks. Radican et al. (2008) reported NHL mortality risks did not differ substantively between infrequent peaks or frequent peaks. Leukemia mortality was not associated with infrequent peak exposure or frequent peak exposure, although the number of deaths in each group (three and nine, respectively) was small. Similarly, mortality from kidney or liver cancers was not associated with infrequent or frequent peak exposure (Table IV).

### 3.5. Acrylonitrile and Lung Cancer

In 1999, IARC reclassified acrylonitrile as possibly carcinogenic to humans (IARC, 1999) based on its determination that an earlier characterization of acrylonitrile as probably carcinogenic to humans (IARC, 1987) was not supported by updated epidemiologic evidence. Lung cancer and prostate cancer were not consistently associated with acrylonitrile exposure in the earliest epidemiologic studies. More recently, the US EPA released a draft IRIS assessment that characterized the weight of evidence for acrylonitrile as likely to be carcinogenic to humans. The risk assessment used an epidemiologic study of the association between cumulative exposure and lung cancer mortality (Blair, Stewart, et al., 1998) for the dose–response assessment to derive an IUR for lung cancer (US EPA, 2011c).

In addition to cumulative exposure, Blair, Stewart et al. (1998) evaluated peak exposure in relation to site‐specific cancer risks for 25,460 workers in eight facilities producing or using acrylonitrile in the United States. Peak exposures were defined as 15‐minute excursions that averaged 20 ppm or greater and the analysis of peaks was based on frequency of peaks. Stewart, Zaebst et al. (1998) conducted an exposure reconstruction for the eight facilities and estimated frequency of peaks based on 8‐hour TWAs that allowed (mathematically) for the possibility of a peak of 20 ppm,^3^ and by considering whether tasks were likely to produce a peak (such as manually taking a process sample). Lung cancer mortality did not increase with cumulative exposure, although lung cancer mortality was slightly increased among the group with the highest cumulative exposure (RR = 1.50, 95% 0.9–2.4 for >8 ppm‐years) when compared to the group with cumulative exposure <0.13 ppm‐years. Lung cancer mortality did not increase with frequency of peaks, and results of the peak analysis for other cancer sites were not presented.

Swaen et al. (2004) evaluated a cohort of 2,842 workers in the Netherlands producing or using acrylonitrile employed at eight companies and 3,961 workers at a nitrogen fixation facility for fertilizer production that were not exposed to acrylonitrile. In earlier years, industrial hygienists had performed exposure monitoring to identify locations with potential for peak acrylonitrile concentrations (see Swaen et al., 1992). The investigators defined peak exposures as “intervals with elevated exposure concentrations in ranges of <10, 10–20, and over 20 ppm which occurred regularly on at least a weekly basis.” The duration of intervals was not specified. Mortality from lung cancer, prostate cancer, brain cancer, and leukemia were analyzed in relation to peak exposure, but no associations were seen.

### 3.6. Ethylene Oxide and LHMs and Breast Cancer

IARC classified ethylene oxide as carcinogenic to humans, based on limited epidemiologic evidence for a causal association with lymphoid tumors (NHL, multiple myeloma, and chronic lymphocytic leukemia) and breast cancer, sufficient evidence in experimental animals, and strong evidence that EO acts by a genotoxic mechanism (IARC, 2008, 2012b). The US EPA (2016) derived an IUR for the combined risk of lymphoid cancer and female breast cancer using an epidemiologic study of mortality risks (and breast cancer incidence) associated with cumulative exposure (Steenland, Stayner, & Deddens, 2004; Steenland, Whelan, Deddens, Stayner, Ward, 2003).

Although Steenland et al. (2004) was one of three epidemiologic studies that evaluated peak exposure metrics in models, the investigators reported that models based on peak exposure did not predict NHL, Hodgkin disease, leukemia, or multiple myeloma, and therefore did not present the results for these models. Steenland et al. (2004, 2003) defined peak exposure as the “highest one‐time exposure.” In an earlier analysis of the cohort, Stayner et al. (1993) reported that the highest one‐time exposure for each employee was the maximum recorded TWA exposure over the worker's job history. Stayner et al. (1993) reported “it was not possible to test the influence of short‐term exposure peaks experienced during the course of the day, since real‐time data on ethylene oxide exposure levels were not available for each individual in the study.” Approximately 2,400 TWA measurements from 1976 to 1985 were used to inform the exposure assessment model (Greife, Hornung, Stayner, & Steenland, 1988). (This highlights the need to review earlier studies to identify a clear definition of “peak” exposure.)

Two additional epidemiologic studies reported “peak” exposures in descriptions of cohorts of ethylene oxide manufacturing workers, but these studies did not evaluate risk estimates using peak exposure metrics (Coggon, Harris, Poole, & Palmer, 2004; Greenberg, Ott, & Shore, 1990). Greenberg et al. (1990) did not define peak exposures but noted that there were no deaths from leukemia “among the subcohort of men who worked where both average and peak exposure levels were probably highest” (likely before 1978 in production units with continuously operating EO converters and recovery systems). Coggon et al. (2004) reported “environmental and personal monitoring carried out since 1977 indicated TWA exposures of less than 5 ppm in almost all jobs, but with occasional peaks of up to several hundred ppm because of operating difficulties in the chemical plants and when sterilizers were loaded and unloaded in hospitals. In earlier years, exposures were probably somewhat higher, and peak exposures above the odor threshold of 700 ppm were reported at both factories and hospitals.” Breast cancer risk was not increased among women hospital workers with continual or intermittent EO exposure (Coggon et al., 2004).

### 3.7. Methylene Chloride and NHL, Biliary Tract, Liver Cancers

An IARC Working Group classified methylene chloride as probably carcinogenic to humans and described the epidemiologic evidence as limited, noting positive associations with cancer of the biliary tract and NHL (IARC, 2017). The US EPA dose–response assessment derived an IUR for the combined risk of liver and lung tumors using a PBPK model of glutathione S‐transferase metabolism dose metrics for mice (US EPA, 2011a).

We identified only one study that discussed peak exposure to methylene chloride. Hearne and Pifer (1999) reported historical ambient measurements ranging between 5 and 100 ppm and short‐term peak concentrations of 1,000 to 10,000 ppm measured during manual adjustments of roll coating machines in cellulose triacetate film manufacture. The investigators also reported peak concentrations greater than 1,000 ppm several times per day when filter media were changed or during the loading of batch mixers in the Dope department; however, there were no risk analyses by a peak exposure metric. Therefore, the literature on methylene chloride contributes to this review only as an example of descriptions of the occurrence of peak exposure (similar to that reported for formaldehyde in several studies).

## 4. ISSUES WITH ESTIMATING AND INTERPRETING ASSOCIATIONS WITH PEAK EXPOSURES IN EPIDEMIOLOGIC STUDIES

There appear to be sound scientific reasons cited for considering peak exposures in epidemiologic evaluations of chemicals carcinogens (Supporting Information Table SII). Not the least of these is the biological understanding that peak exposures that exceed certain levels or thresholds—even for brief periods of time—likely trigger metabolic processes or direct genetic, epigenetic, or cytotoxic damage that lead to cancers (Kriebel, Checkoway, & Pearce, 2007; Macaluso et al., 2004; Morgan et al., 1998; Rappaport, 1991; Stewart et al., 1986, 1992). In theory, accurate characterization of these peak exposures would enhance the identification of exposed groups with increased cancer risks and their differentiation from groups at lower or no increased risk. Identification of risks associated with peak exposures also would impact the risk assessment process and derivation of exposure limits—especially occupational STELs.

However, we identified surprisingly few, if any, good examples where efforts to define and evaluate peak exposures systematically and rigorously have informed valid risk assessment. Our review points to several limitations and issues that might, at least in part, explain this dearth of epidemiologic studies effectively identifying increased cancer risks among those exposed to peaks.

### 4.1. Lack of Consistent Definitions of Peak Exposures

Epidemiologic studies sometimes rely on data—largely 8‐hour TWA measurements—routinely collected to demonstrate compliance with regulatory standards. “Peak exposure” is not a standard industrial hygiene term, however, and may not be measured even when a STEL exists for a chemical. In the epidemiologic literature we reviewed, peak exposure was defined in different ways, including but not limited to the following: the highest short‐term (15‐minute) exposure concentration sustained by an individual (Hauptmann et al., 2009), the highest TWA experienced by an exposed individual (Steenland et al., 2003, 2004), the number of days with peaks that exceed an estimated short‐term value (Collins et al., 2003), the estimated relative peak and estimated number of peaks (Beane Freeman et al., 2009), and the estimated number of total peaks exceeding a threshold of exposure (>100 ppm butadiene or >50 ppm styrene), regardless of duration (Cheng, et al., 2007; Graff et al., 2005; Macaluso et al., 2004).

Others relied upon derived semiquantitative peak scores or qualitative rating (Blair, Hartge, et al., 1998; Morgan et al., 1998; Stenehjem et al., 2015). One study evaluated effect of peaks by combining “peak” with cumulative exposure: exposed to low or medium cumulative dose without peaks, low or medium cumulative dose with peaks, high cumulative dose without peaks, and high cumulative dose with peaks (Charbotel et al., 2006). Finally, Graff et al. (2005) also analyzed leukemia risks separately by butadiene ppm‐years that had been partitioned into exposure concentrations above and below the peak threshold (i.e., >100 ppm butadiene) (Graff et al., 2005; Macaluso et al., 2004).

Some definitions of peak exposure incorporated a time dimension, such as a 15–60 minute excursion above an average or some threshold for a specified frequency of occurrence (e.g., peaks occur at least weekly) (Checkoway et al., 2015; Glass et al., 2014; Schnatter et al., 2012). Others included “short‐term exposure levels” (without specifying the time interval). The definitions of peak varied across the epidemiologic literature, which complicates interpretation of data from studies applying different peak metrics. Thus, uniform definitions for peak, relevant to research on environmental risk factors for cancer, clearly are needed before their ability to improve risk assessments can be determined.

### 4.2. Lack of Reliable Exposure Data or Validated Peak Metric

In the studies that analyzed risks in relation to some form of peak exposure, nearly all peak exposure estimates were based on expert judgment and not on measurements. Although study investigators frequently discussed the validity and reliability of the summary exposure estimate (e.g., average estimates in the JEM cells), the validity and reliability of peak exposure metrics were rarely considered. Validation of peak exposures was not reported in any study, although the U.S. formaldehyde industrial workers cohort reported an evaluation of the TWA measurements. Blair and Stewart (1990) also reported correlations among various formaldehyde exposure metrics; however, this would not necessarily validate any of the metrics. Short‐term exposure measurements rarely were available, and in none of the studies were actual peaks measured for individual workers or for job‐defined groups of workers applied to a JEM. This is not surprising, as most studies relied on average exposure or qualitative exposure metrics based on job title/work area and calendar year(s). Although some studies had limited historical industrial hygiene measurements, few can be considered good indicators of individual worker or group‐level peak exposure. Average exposures assigned to workers with higher and lower actual exposures leads to misclassification. Assuming a true relationship with peak exposures, this random misclassification would be expected to reduce the relative risk estimates associated with peak exposures and the associated risk numbers.

In many cases, the examples we found in relation to peak exposure did not report sensitivity analyses of the potential magnitude and direction of bias associated with the use of the peak exposure metric. However, some studies conducted analyses that evaluated two measures of peak exposure, such peak intensity and frequency (hourly, daily, weekly, monthly) or number of peaks, or hybrid measures with a peak component (e.g., peak frequency, average or cumulative peaks). For example, Beane Freeman et al. (2009) evaluated the frequency of peaks and reported relative risks of any LHM did not increase with the number of peaks ≥ 4 ppm, although the data were not shown. Even if this approach reasonably describes the exposure of groups of employees working in a given job or area, it is not known whether any specific individual (including those developing cancers) actually had been exposed to any peaks. In contrast, there was fairly good agreement between risk estimates for leukemia associated with ppm‐years above exposure intensities of 100 ppm butadiene and number of total peaks of butadiene above 100 ppm (Graff et al., 2005).

In the benzene literature, risks were elevated for peak and for cumulative exposure. In contrast, the styrene example did not find relations between peak exposure and any of the LHMs. Beane Freeman et al. (2009) reported associations between their peak formaldehyde exposure metric and LHM and ML, but not with cumulative, average, or duration of exposure—or with frequency (hourly, daily, weekly, monthly, no peaks) or total number of peaks. Hauptmann et al. (2009) found no association between peak exposure and ML; however, when the analysis of “peak exposure” was restricted to embalmers, a statistically significant trend was seen with peak exposure among embalmers for whom a lifetime history of performing more than 500 embalmings was reported by next of kin (many decades later). The odds ratios for the different peak exposure categories were based on few myeloid leukemias and even fewer AMLs, and none were statistically significant.

Further complicating this is the likelihood that peak exposures occurred more frequently in workplaces in the distant past, and their frequency (and possibly magnitude) has declined over time due to improved controls and regulations. For example, although increased AML risk was reported among embalmers, 76% of those whose cause of death was classified as AML were first employed in a funeral home prior to WWII, and 44% were first employed before 1932, requiring speculative approaches to exposure assessment (in which peak exposures ultimately were reported in categories defined by the following narrow ppm ranges: <7; >7–9.3; >9.3) and reduced comparability with the control group (Hauptmann et al., 2009).

Therefore, while epidemiologially evaluating cancer risks assocated with peak exposure remains conceptually atrractive, there are too few examples where studies demonstrate the contribution of peak exposure to risk beyond that estimated—even if poorly—by other exposure metrics.

### 4.3. Exposure Misclassification

**Figure 1:**
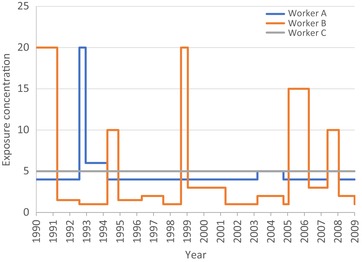
Peak exposure profiles for three workers with identical cumulative exposure after 20 years. Worker A encounters one peak, Worker B encounters five peaks, and Worker C does not encounter peaks.

Peaks and their duration are also components of average and cumulative exposure. However, as illustrated in Fig. 1, variability of exposure intensity (including short‐term excursions or exceedances that may be considered exposure peaks) and frequency over time can result in distinctly different exposure profiles for workers with the same cumulative exposure. Epidemiologically, this would be considered exposure misclassification. Sufficient exposure sampling is necessary to capture relatively rare occurrences of peaks; otherwise, they will be missed and contribute only trivially to cumulative exposure estimates. When time‐varying exposures fluctuate from hour to hour and day to day between cohort members in the same job setting, infrequent sampling of exposure risks missing the measurement of peak exposures because these peak exposures may fall outside of rare sampling occasions. These peak exposures may constitute a large fraction of cumulative exposure, in theory. In addition, historical sampling data were often collected to demonstrate compliance with a regulatory standard rather than to identify and characterize heterogeneity in exposure scenarios. This practice likely results in exposure misclassification even when the exposure metric is cumulative exposure.

Many studies offered that nondifferential misclassification of cumulative exposure results in risk estimates that are biased toward the null (Beane Freeman et al., 2009; Hauptmann et al., 2009; Radican et al., 2008; Schnatter et al., 2012; Stenehjem et al., 2015). Although seemingly true, Jurek, Greenland, Maldonado, and Church (2005) and Lash et al. (2014) suggest that nondifferential exposure misclassification only guarantees an expectation that *on average*, the bias will be toward the null when exposure is assessed using a dichotomous variable (exposed/not exposed). It is well known that when risks are evaluated using multiple exposure categories (as they generally are in historical reconstructions of average and peak exposure intensity), exposure misclassification results in biased risk estimates in the middle categories that are away from the null (Dosemeci, Wacholder & Lubin, 1990; Jurek et al., 2005; Weinberg, Umbach, & Greenland, 1994). Furthermore, the bias in any particular study can be away from the null because the actual misclassification can result in a data arrangement far from what is expected. We raise one particular concern with respect to this issue: in studies that rely upon expert judgment to estimate average exposure intensity and peak exposure (especially semiquantitative and quantitative measurements) based on job tasks and industrial processes, it is conceivable (even rational) that experts are subject to an “anchoring effect,” a cognitive bias in which initial information (estimates of or measurements of TWA concentrations) may influence the estimate of peak exposure. For example, experts may be more likely to assign probability and/or frequency of experiencing peak exposures when the 8‐hour TWA value (measured or estimated) is high and may be less likely to assign the probability and/or frequency of experiencing peak exposures when the 8‐hour TWA value is low. Conversely, experts may be more likely to estimate higher 8‐hour TWA values when information about processes and job tasks suggests higher probability and/or frequency of peak exposure. When peak is defined by multiple categories, which are themselves based on consideration of multiple categories of average concentrations, we hypothesize that estimated peak exposure in particular is susceptible to increased variability and bias that is not predictable as to its direction or magnitude. In addition, analyses by multiple exposure metrics also increase the opportunity for false‐positive associations to arise simply by chance (Blair, Stewart, et al., 1998).

### 4.4. Consideration of Disease Latency in Relation to Peak Exposure Metric

Specific types of most cancers, including the LHMs, are relatively rare, and all but the largest occupational epidemiologic studies have too few cases to allow a meaningful analysis across peak exposure categories. If peak exposures also are rare, the number of cases with peak exposures will be even smaller, precluding any meaningful estimate of relative risk. For example, Checkoway et al. (2015) noted that the updated NCI formaldehyde industrial workers study (Beane Freeman et al., 2009) identified 34 AML deaths, the type of leukemia most plausibly related to chemical exposures. However, less than one‐third (*n* = 13) of these were classified as having had any peak exposure (defined as working in jobs expected to have peak formaldehyde exposures >2 ppm).

Furthermore, when reasonable latency time windows are considered, even fewer observed cases remain plausibly linked with exposure. Leukemias likely have shorter latencies than solid tumors. Studies of atomic bomb survivors in Japan reported the greatest increases in AML incidence occurred 5–7 years after exposure and declined thereafter (Darby, Nakashima, & Kato, 1985; Moloney & Lange 1954). Incidence of AML following chemotherapy is highest within 5–10 years after treatment (Deschler & Lubbert, 2006) but could be as short as 2–3 years following treatment with topoisomerase inhibitors (IARC, 2012a). Considering latency in the Beane Freeman et al. study (2009) of 13 AML deaths among workers classified as having a peak exposure, only four had jobs associated with peak exposure within the 20 years preceding death, and only one of these occurred within a conservative latency window of 2 to 15 years after the last peak exposure. Therefore, it is not possible to draw any conclusion regarding peak exposure and AML from these study data (Checkoway et al., 2015).

Many epidemiologic studies address cancer latency by evaluating different time windows of exposure; for example, Glass et al. (2014) used a 2–20‐year exposure window and Schnatter et al. (2012) used a 2–15‐year exposure window. Relevant time windows of risk may be evaluated by lagging cumulative (or other) exposures, and only exposures that occurred in specified time intervals before diagnosis or death are considered. Only a few studies that reported cancer risks in relation to peak exposure metrics also conducted analyses in which the investigators lagged peak exposures (Beane Freeman et al., 2009; Checkoway et al., 2015; Cheng et al., 2007; Hauptmann et al., 2009; Steenland et al., 2003, 2004).

Furthermore, even for agents classified as leukemogens (benzene, formaldehyde, BD), which we assume have shorter latencies, we found little compelling evidence that any peak exposure metric was predictive of increased leukemia (or LHM) risk, and especially in the absence of demonstrable increased risks with cumulative exposure. This likely reflects weaknesses and inconsistencies in the exposure metrics employed, even if an association with peak exposure exists.

Some modern exposure monitoring approaches—even if not yet commonly used—would allow rapid or even real‐time measurement of air concentrations. In theory this would validly identify the exact time and magnitude of peak exposures; however, detection of increased cancer risk as a consequence of these exposure(s) would require (1) large numbers of workers exposed to peaks and (2) the workers to be followed for up to 10 or 15 years due to latency issues. Therefore, it is unlikely that studies using measured peak exposures to examine cancer risks will be common if conducted at all, and uniform and transparent approaches will need to be refined for retrospectively deriving peak exposure metrics for the relevant latency time window.

### 4.5. Considerations of Biological Processes and Mechanisms Associated with Peak Exposure

We postulate that it is plausible that one or more mechanisms for carcinogenesis may be initiated by a sudden upward spike in the intensity of exposure, to the degree that such an exposure reflects an underlying “dose rate” that suddenly accelerates. Do contributions to risk differ if such a “dose rate” plateaus and moves to a higher average exposure or, alternatively, decreases?

Specific hypotheses regarding the importance of patterns of exposure are rarely discussed explicitly (i.e., how does the pattern of exposure factor into the increased risk?). For example, alternative explanations for increased risks of neoplasms at low levels of exposure include: (1) average exposures in the past were underestimated based on recent lower levels of exposure and past exposures were actually considerably higher than estimated; and (2) occasional high exposures (peaks) factor disproportionately into the risks associated with lower average exposures. Instead, pattern of exposure is viewed as part of the black box—and, therefore, risk estimates should be calculated using multiple exposure metrics because the underlying disease process is unknown. Interestingly, cumulative exposure has emerged as the default metric in a majority of epidemiologic studies that quantify exposures, most likely due to simplicity (e.g., “more exposure should result in more risk”) rather than a biological understanding of underlying disease pathways and what type of exposures (e.g., peaks or other exposures exceeding some threshold) increase risk.

## 5. CHALLENGES FOR IMPLEMENTATION OF UNIFORM PEAK EXPOSURE METRICS FOR CANCER RISK EVALUATION

For several chemicals classified as known or probable carcinogens, we attempted to identify the main epidemiologic studies that reported relative risks in relation to some form of peak exposure. However, we found “peak” exposure characterization to be highly idiosyncratic. Therefore, perhaps it is not surprising that we did not find any clear or convincing examples in the epidemiologic literature of increased cancer risks in relation to peak exposure estimates for chemicals, even where the chemicals had been classified as known carcinogens based on or strongly influenced by the epidemiologic evidence. To our knowledge, no regulatory carcinogenic risk assessment has been based on epidemiologic findings using peak exposure. The draft IRIS assessment for formaldehyde reported an association between peak exposure and LHM, especially myeloid leukemia, but relied on cumulative exposure metrics in the dose–response assessment. All of the studies we reviewed were limited by the simple fact that industrial hygiene measurements of excursions in short‐term exposures are relatively rare, and there are no standard sampling schemes to measure them. Most studies classified workers as having peak exposures based on expert opinion or an understanding of the working environment and not specific exposure measurements. Therefore, real‐time monitoring of workplace air quality may shed some light on this conundrum; however, dose–response modeling based on peak exposure has not been defined and will require development.

Standardized exposure definitions for “peak exposure” do not exist currently in epidemiology but warrant separate consideration given possibly unique contributions to risk. Unlike cumulative exposure measures, which can be compared readily across studies, peak exposure has not been defined sufficiently similarly across studies measuring the same outcomes. Therefore, it is not clear how the contribution to risk from peak exposure can be evaluated separately from cumulative exposure in elucidating exposure responses for risk assessment and risk assessors will not be able to evaluate contributions to risk specifically associated with peak exposure.

Although it is not within the scope of this article to derive standard definitions for peak exposure, a number of parameters and desirable qualities can be offered. First, quantified exposure measurements and estimates (e.g., ppm) are preferable over qualitative estimates (e.g., exposed/not exposed, or ranked on an ordinal scale, such as “low/background,” “moderate,” and “high”), provided that they are reasonably valid and some estimate of variability (including measurement error) is included. Second, the time period and frequency for which a peak exposure estimate is likely to be valid should be determined, as the time dependency of exposure to carcinogens can be important (and not limited to latency issues). Third, potential for individual workers’ peak exposures to differ from group values should be addressed, as it is conceivable that environments likely to confer peak exposures are not uniformly likely to do so across work locations, times, and workers. Fourth, and especially where estimates are less data derived and more assumption laden, sensitivity testing should be used to determine the influence of various components to the peak metric. In circumstances of limited data or poor understanding of the relevant underlying carcinogenic mechanisms, presenting more detailed documentation and explanation of rationale are helpful, and will clarify situations that are hypothesis driven from those that are more exploratory.

Choice of peak metric likely matters. There are multiple variations of time‐varying exposure patterns consistent with peak exposures. One potentially useful way to consider peak is under two exposure circumstances: (1) peaks are commonly encountered (extreme values are frequent) or, conversely, (2) few peaks are encountered such that extreme values are rare.

Given that occupational exposure concentration(s) often vary considerably over time, a basic probabilistic approach to defining peak exposure in these terms might serve as a reasonable starting point. The simplest definition of peak exposure, therefore, might be an exposure event in which the measured or estimated concentration exceeds a specified exposure threshold value. This alone, however, does not address the duration of time over which this concentration level is exceeded, the magnitude of the peak, or the number of exceedances. Alternatively, as the pharmacokinetics of a potential carcinogen are better understood, an effective dose can be estimated and from this—and somewhat ideally—an equivalent peak exposure derived. The pharmacokinetically derived peak conceptually is not limited to the single dimension of exposure concentration (expressed above as exceedance of an arbitrary percentile) but can combine concentration and time to define a target dose. By extension, an effective dose rate might also be derived.

A thorough analysis of potential etiologic relations would involve estimation of risks associated with: peaks based on various thresholds; magnitude of peaks above thresholds; number of peaks; and timing of peaks in relation to disease onset or diagnosis. These approaches can take the form of sensitivity analyses comparing findings based on various peak exposure metrics. Assessing the sensitivity analysis findings in relation to chemical‐specific carcinogenesis models derived from toxicological research can enhance data interpretation.

Assuming uniform approaches for developing standard peak exposure metrics are successful, the dose–response assessment still requires careful consideration. In particular, when peak exposure metrics (and not cumulative exposure) drive epidemiologic cancer hazard determination, the dose–response assessment should not be based on cumulative exposures. IURs inherently characterize cancer risks as linear dose–response relations over the entire exposure spectrum, typically reflecting cumulative exposure. New risk metrics are needed to describe risks that are not monotonic, including risks that increase in response to peak exposures. Regardless of how peaks are defined, they inherently argue against the “additivity to background” or “every molecule counts” perspective that is synonymous with risk as a direct function of cumulative exposure. This acknowledges that risk will be more accurately quantified when the most biologically relevant characterization of exposure is captured.

Contrasts of associations observed for peaks versus those observed for cumulative exposure and exposure duration are highly recommended, especially as there are clear implications for risk assessment. Although peak exposure correlates with other exposure metrics, selection of peak exposure as the primary exposure metric of interest should be accompanied by judicial consideration of other metrics in attempting to isolate and characterize causal exposure characteristics. It should be appreciated that, by definition, peak exposure contributes to cumulative exposure. As such, a correlation between dose–response models derived from peak and cumulative exposures should be anticipated in many instances, and the biological rationale—as well as results of sensitivity analyses—should guide the selection and interpretation of the best exposure metric and subsequently improve the derivation of risk numbers including the IUR and others not assumed to be linear. Future epidemiologic research, including studies of new cohorts and reanalyses of data from existing cohorts, ideally will address and contrast dose–response associations for cumulative and peak exposures, where data permit suitable exposure assessment. However, investigators should bear in mind that exploring associations using multiple exposure metrics will increase the number of chance (i.e., false‐positive) results, and unless there is an underlying biological basis for interpreting them as causal, they should be viewed with caution.

## Supporting information


**Table SI**. Uses, Exposure Limits, Cancer Characterization, Inhalation Unit Risk, and Epidemiologic Studies with Quantitative Exposure Assessment Metric by Substance
**Table SII**. Rationale for Using Peak Exposure or Reason for Considering Peak Exposure as Reported by Study Investigators

## Footnotes

1 See list of classifications by cancer site at https://monographs.iarc.fr/agents-classified-by-the-iarc/.

2 See https://www.epa.gov/iris.

3 That is, a minimum 8‐hour TWA of about 0.6 ppm (20 ppm × 15 min/480 min) (Stewart, Zaebst et al., 1998).
