# Supplementary material for: Peak Exposures in Epidemiologic Studies and Cancer Risks: Considerations for Regulatory Risk Assessment
Source: Risk Anal. 2019 Mar 29;39(7):1441–64. doi: 10.1111/risa.13294 (PMC6850123; doi:10.1111/risa.13294)
Supplement: Supplementary file 1 — Table SI. Uses, Exposure Limits, Cancer Characterization, Inhalation Unit Risk, and Epidemiologic Studies with Quantitative Exposure Assessment Metric by Substance Table SII. Rationale for Using Peak Exposure or Reason for Considering Peak Exposure as Reported by Study Investigators [file RISA-39-1441-s001.docx]

**Supplementary Table 1**. Uses, exposure limits, cancer characterization, inhalation unit risk and epidemiologic studies with quantitative exposure assessment metric by substance

| **Substance / Uses** | **Exposure limits** | **Strength of Evidence characterization (IARC)** | **Inhalation Unit Risk, dose-response assessment (US EPA)** | **Epidemiologic studies with exposure assessment considered in strength of evidence characterization *** |
| --- | --- | --- | --- | --- |
| **Acrylonitrile**  Monomer used as an intermediate in the production of acrylic and modacrylic fibers, acrylonitrile butadiene styrene resins, styrene acrylonitrile resins, and other chemical intermediates | PEL 2 ppm 8-h TWA  C 10 ppm  TLV 2 ppm 8-h TWA | Possibly carcinogenic to humans (IARC, 1999, Vol 71)  Likely to be carcinogenic to humans based on animal evidence (EPA DRAFT 2011a)  (Blair et al., 1998) | 6.8 x10^-5^ per µg/m^3^  (EPA, 1987)  Respiratory cancer in humans via inhalation (O’Berg 1980),  Extrapolated from a relative risk model adjusted for smoking, based on continuous lifetime equivalent of occupational exposure.  3.3 × 10^-5^ per µg/m3  (life time risk adjusted for early life susceptibility) (EPA DRAFT, 2011a)  Dose-response assessment: Lung cancer mortality based on cumulative exposure (Blair et al., 1998) | Benn & Osborne, 1998  **Blair et al.,** 1998  Chen et al., 1987  Collins et al., 1989  Marsh et al. 1999, 2001  O’Berg et al., 1985  **Swaen et al., 2004, 1998, 1992**  Symons et al., 2008  Wood et al., 1998 |
| **Benzene**  Historically, as an industrial solvent | PEL 1 ppm (3.2 mg/m^3^) 8-h TWA  TLV 0.5 (1.6 ppm mg/m^3^) 8-h TWA  STEL 2.5 ppm (8 mg/m^3^) | Carcinogenic to humans (Loomis et al., 2017)  Causes ANLL including AML (IARC, 1982; 2012; Loomis et al., 2017)  Limited evidence for CLL, multiple myeloma, NHL (small minority of Working Group concluded that evidence was sufficient for NHL) | 2.2 x 10^-6^ to 7.8 x 10^-6^ per µg/m^3^ (6.9 x 10^-7^ to 2.4 x 10^-6^ per ppm) (EPA, 1998)  Linear low-dose extrapolation below range of observation using maximum likelihood estimates.  Cumulative exposure to benzene and leukemia mortality data from epidemiology (the Pliofilm cohort (Crump 1994; Crump & Allen 1984; EPA 1998; Paustenbach, Bass, & Price 1993; Rinsky et al., 1987; Rinsky, Young, & Smith 1981) | Bloemen et al., 2004  **Collins et al.,** 2003  **Glass et al.,** 2014; 2003  Guénel et al., 2002  Hayes et al. 1996; 1997  Linet et al., 2015  Lewis et al., 2003  Raaschou-Nielsen et al., 2001 (ca-co)  Rinsky et al., 1981; 1987; 2002  Rushton & Romaniuk, 1997  **Schnatter et al.,** 1996; 2012  Seidler et al., 2007 (ca-co)  **Stenehjem** et al., 2015  Wong 1987; 1995  Wong, Harris, & Smith 1993  Yin et al., 1996 |
| **1,3-Butadiene**  Used as an intermediate and a monomer in the manufacture of polymers, such as styrene-butadiene rubber, poybutadiene rubber, polychloroprene and nitrile rubber | PEL 1 ppm (8-h TWA)  STEL 5 ppm (15 minutes)  TLV 2 ppm (4.4 mg/m^3^) (8-h TWA) | Carcinogenic to humans (IARC, 2012)  IARC Working Group described epidemiologic evidence as “limited for a causal association with LHMs, specifically NHL, multiple myeloma, and chronic lymphocytic leukemia.”  US EPA describe epidemiologic evidence as “sufficient.” | 3 x10^-5^ per µg/m^3^  (EPA, 2002)  Based on epidemiologic studies (Delzell et al. 1995; Health Canada 2000)  Derived using lifetable analysis with leukemia incidence data and applying an adjustment factor of 2 | **Cheng et al**., 2007  Delzell et al., 2001, 2006  Divine & Hartman 2001  Graff et al., 2005  Macaluso et al., 1996  Matanoski et al., 1997 |
| **Ethylene oxide**  Used as a sterilizing agent, and as an intermediate in the production of ethylene glycols and other chemical derivatives. | PEL 1 ppm (8-h TWA)  STEL 5 ppm (15 minutes)  TLV 1 ppm (1.8 mg/m^3^) (8-h TWA) | Carcinogenic to humans (IARC, 2012; 2008)  Limited epidemiologic evidence for EO and NHL, multiple myeloma, and chronic lymphocytic leukemia; strong evidence that EO acts by a genotoxic mechanism. | 3 x10^-3^ per µg/m^3^ (EPA, 2016)  Combined risk of lymphoid cancer and female breast cancer  Linear extrapolation using a two-piece linear spline model.  Dose-response assessment: Lymphoid cancer, (female) breast cancer using cumulative exposure (Steenland et al., 2003, 2004) | Coggon et al., 2004  Greenberg, Ott, & Shore 1990  Hogstedt, Aringer, & Gustavsson 1986  Morgan et al., 1981  **Steenland et al.,** 2003, 2004  Swaen et al 2009  Valdez-Flores et al., 2010 |
| **Formaldehyde**  Commonly used in production of other chemicals and products, including building materials, coatings, fabric treatments, and cosmetic | PEL 0.75 ppm 8-h TWA  STEL 2 ppm  TLV 0.1 ppm 8-h TWA  STEL 0.3 ppm | Carcinogenic to humans (IARC, 2006; 2012)  Causes myeloid leukemia and nasopharyngeal cancer (IARC, 2006; 2012)  Small majority of Working Group concluded evidence was sufficient for ML. | 8.1 × 10^–2^ per ppm (6.6 × 10^–5^ per μg/m3) [EPA DRAFT, 2010 Toxicological Summary] (EPA, 2010)  Linear low dose extrapolation below range of observation using maximum likelihood estimates. Used cumulative exposure from epidemiology for NPC, Hodgkin lymphoma and leukemia (Beane Freeman et al., 2009) | **Beane Freeman** et al., 2009, 2013  Blair et al., 2001 (ca-co  **Hauptmann et al.,** 2009 (ca-co)  Hildesheim et al., 2001 (ca-co)  Laforest et al., 2000  Luce et al., 1993 (ca-co)  Marsh, Youk, Stone, et al., 2001  Partanen et al., 1993 (ca-co)  Tarvainen et al., 2008)  Vaughan et al., 2000 (ca-co)  Wang et al., 2009 (ca-co)  West et al., 1995 (ca-co)  Youk et al., 2001)  **More recently published**  Coggon et al., 2014  **Checkoway et al.,** 2015  Marsh et al., 2016  Meyers, Pinkerton, & Hein 2013  Pira et al., 2014  Saberi Hosnijeh et al., 2013  Talibov et al., 2014 |
| **Methylene chloride**  Solvent used in paint strippers, aerosol propellants, as a process solvent in pharmaceuticals, in metal degreasing.  Also used to produce cellulose fiber, photographic film, and in production of pesticides and refrigerants | PEL 25 pppm (8-h TWA)  STEL 125 ppm  TLV 50 ppm (174 mg/m^3^) (8-h TWA) | Probably carcinogenic to humans (IARC, 2017  Limited evidence of cancer of the biliary tract and NHL in humans | 1 x10^-8^ per µg/m^3^ (EPA, 2011b)  Combined risk of liver and lung tumors (EPA, 2011b)  Dose-response assessment based on modified mouse PBPK model using GST metabolism dose metrics for mice | Gibbs, Amsel, & Soden 1996  Hearne & Pifer 1999  Lanes et al., 1993  Ott et al., 1983  Radican et al., 2008(Radican, Blair, Stewart, & Wartenberg 2008)  Tomenson 2011  Tomenson 1997 |
| **Styrene**  Monomer used as an intermediate in the production of polymers, reinforced plastics, and styrene-butadiene rubber industry | STEL 40 ppm (170 mg/m^3^) | Probably carcinogenic to humans (Kogevinas et al. 2018)  Limited evidence in humans: LHMs, myeloid leukemia (most informative study: Christensen et al., 2018)  Sufficient evidence in animals | IUR has not been derived | Christensen et al., 2018  Collins, Bodner, & Bus 2013  Coggon et al., 2015  Delzell et al., 2001  Graff et al., 2005  Kogevinas et al., 1994  Nissen et al., 2018  Sathiakumar et al., 1998 |
| **Trichloroethylene**  Used historically as a metal degreasing agent in the aerospace and other industries. Also used historically in processing food, as an anesthetic and in dry cleaning.  TCE has been found in consumer products: automotive products, paints and coatings, cleaners, and polishes. | PEL 100 ppm 8-h TWA  C 200 ppm  STEL 300 ppm (single period up to 5 minutes in any 2 hrs)  TLV 10 ppm (54 mg/m^3^) 8-h TWA  STEL 25 ppm (135 mg/m^3^) | Carcinogenic to humans (IARC, 2014)  Causes cancer of the kidney.  Positive association between TCE and NHL also reported | 4.1 x10^-6^ per µg/m^3^  (EPA, 2011c)  Renal cell carcinoma, non-Hodgkin's lymphoma (NHL) and liver cancer risks combined  Used cumulative exposure from epidemiology (Charbotel et al., 2006; EPA, 2011c; Raaschou-Nielsen et al., 2003) | Anttila et al., 1995  Bahr et al., 2011  Boice et al., 1999  Bruning 2003 (ca-co)  Christensen et al., 2013 (ca-co)  **Charbotel et al**., 2006 (ca-co)  Cocco et al., 2010 (ca-co  Gold et al., 2011 (ca-co)  Lipworth et al., 2011  **Morgan** et al., 1998)  Moore et al., 2010 (ca-co)  Pesch et al.,2000 (ca-co)  Purdue et al., 2011)  Raaschou-Nielsen et al., 2003  **Radican et al.,** 2008  Seidler et al., 2007 (ca-co)  Vamvakas et al., 1998 (ca-co)  Zhao et al. 2005) |

*Bold font indicates epidemiologic studies that reported relative risks in relation to peak exposure metrics. Other studies listed here were cited by the IARC as informing the strength of evidence and reported relative risks in relation to quantitative and semi-quantitative exposure metrics, or a high level qualitative exposure assessment (such as probability of exposure as high/medium/low). Epidemiologic studies that reported only surrogates or simple indicators of exposure are not listed here.

**Supplementary Table 2**. Rationale for using peak exposure or reason for considering peak exposure as reported by study investigators

| **Chemical** | **Rationale / Consideration for using peak exposure in analysis** |
| --- | --- |
| Acrylonitrile | - “The opportunity to **evaluate disease risk by different exposure metrics is particularly valuable in studies such as ours, where no clear exposure-response relationship was observed**. Although acrylonitrile is an animal carcinogen [the metabolite 2-cyanoethylene oxide is believed to be the ultimate carcinogen (38, 39)] and it can form DNA adducts, many details regarding the mechanism of action in humans are not well understood. **When it is not clear which exposure measure is the best surrogate for delivered dose, the ability to evaluate risks by different exposure types increases confidence that an association has not been missed simply due a reliance on an inappropriate surrogate**. The value of using several exposure measures is underscored by the finding **that different exposure metrics often classify workers differently**. This finding has been reported in other studies (Blair et al., 1990 [*formaldehyde* Jarup, Pershagen, & Wall, 1989 [*arsenic*]), and it occurs in this investigation of acrylonitrile.” (*Blair et al., 1998*) - “Although analysis by several different measures of exposure diminishes the chances of missing an association, it **increases the opportunity for chance excesses because of the larger number of comparisons**.” (*Blair et al., 1998*) - “Several exposure metrics were developed in addition to the TWA 8h estimates to provide the opportunity **to investigate risks from disease mechanisms other than cumulative exposure**.” (*Stewart et al., 1998, exposure assessment for Blair et al., 1998).* - “On the other hand, **increased risk might be associated with peak exposure rather than continuous low-level exposure** and may be confounded by the exposure to other carcinogens in the workplace.” (*Swaen et al., 1992, discussing exposure assessment used by Swaen et al., 1992; 2004*). |
| Benzene | - “The dose rate of benzene, or the exposure concentration received over time, appears important for assessing cancer risk in experimental systems. Specifically, **some argue that a critical concentration must be present before cancer risk is increased**.” *Collins et al., 2003.* - “…**to capture different aspects** of benzene exposure.” *Schnatter et al., 2012.* - “A non-linear relationship between total dose and effect is suggested by pharmacokinetic models (Bois and Paxman, 1992), from consideration of the probable genetic mechanisms (API, 1996) and from toxicological data (Ricci and Cox, 1997). **It appears that long exposure to low levels of benzene is not equivalent in risk to short high exposures**.” *Glass et al., 2000.* - “Although the link with LH cancers has been studied thoroughly in recent years, **there is limited evidence to decide which characteristics of benzene exposure are the most relevant for identification and assessment of LH cancer risk**: for example, cumulative exposure, exposure duration, intensity, or peak exposures (Collins et al, 2003).” *Stenehjem et al., 2015* |
| 1,3-Butadiene | - “The main objective in developing the task exposure estimates was to characterize the likely average exposure conditions. To the extent feasible, however, we estimated variability in exposure. In particular, we often included known variability in the frequency and duration of performance of a specific task in the calculation of peak exposure frequencies and of time-weighted average exposure estimates.” *Delzell* *et al., 1995* |
| Ethylene oxide | - **Rationale not explicitly stated**: “We also tried models using peak exposure, average exposure, and duration of exposure, with no lag or different lags.” *Steenland et al., 2004* - **Rationale not explicitly stated**: “Although cumulative exposure is generally the exposure metric of primary interest in epidemiologic studies, estimates of duration of exposure, average time-weighted exposure level, and the maximum time-weighted exposure level were also evaluated in the analyses.” *Stayner et al., 1993* |
| Formaldehyde | - “Laboratory studies suggest that **peak exposure to formaldehyde may be more damaging to biologic tissue than cumulative exposure**.” *Stewart et al. 1986 (exposure assessment for Bean Freeman et al., 2009)* - “…one of these studies *[NCI]* **used quantitative exposure estimates and observed an association for peak formaldehyde exposure**, which is consistent with formaldehyde being a causative agent for LHMs.” *Hauptmann, et al., 2009.* |
| Methylene chloride | - Peak exposures were described in a narrative, and risks were not presented by peak exposure metric. |
| Styrene | - “Short (<1 h) and long (1–8 h) term measurements were examined separately using statistical analyses. The reason for such segregation of the data was **that measurements that lasted <1 h were more likely to cover only one task** (lamination) **where the highest exposures were expected**, whilst the measurements that spanned longer time periods were likely to encompass several tasks, and thus are more representative of exposure concentrations averaged over the full duration of a work-shift.” *Kolstad, Sonderskov, & Burstyn, 2005* (exposure assessment for Christensen et al. 2017) |
| TCE | - “We used “peak” exposure to evaluate hypotheses that **high-level exposure involves different metabolic pathways and may, thus, be more important in assessing cancer risk**.” *Morgan et al. 1998* - “…a slightly elevated rate ratio was found in a study on the mortality of aerospace workers exposed to TCE (*Morgan et al., 1998*). **In those with peak exposures at medium and high levels,** the rate ratio was 1.89 (0.85–4.23).” *Charbotel et al., 2006* - “[The exposure assessment of TCE] provides the opportunity to evaluate potential effects of continuous and peak exposures. Either or both could be important in cancer mortality; for example, if cumulative exposures are critical, adverse health effects could result from relatively continuous exposure at low levels. **Conversely, if adverse health effects occur only after exposure to a particular level, workers who have had peak exposures may be at increased risk**.” *Stewart et al., 1991 (exposure assessment for Radican et al., 2008* |

**REFERENCES IN SUPPLEMENTARY TABLES**

Anttila, A., Pukkala, E., Sallmen, M., Hernberg, S., & Hemminki, K. (1995). Cancer incidence among Finnish workers exposed to halogenated hydrocarbons. *Journal of Occupational and Environmental Medicine, 37*(7), 797-806.

Bahr, D. E., Aldrich, T. E., Seidu, D., Brion, G. M., Tollerud, D. J., Paducah Gaseous Diffusion Plant Project, T., . . . & Minor, S. (2011). Occupational exposure to trichloroethylene and cancer risk for workers at the Paducah Gaseous Diffusion Plant. *International Journal of Occupational Medicine & Environmental Health, 24*(1), 67-77. doi: 10.2478/s13382-011-0007-1

Beane Freeman, L. E., Blair, A., Lubin, J. H., Stewart, P. A., Hayes, R. B., Hoover, R. N., & Hauptmann, M. (2009). Mortality From Lymphohematopoietic Malignancies Among Workers in Formaldehyde Industries: The National Cancer Institute Cohort. *Journal of the National Cancer Institute, 101*(10), 751-761.

Beane Freeman, L. E., Blair, A., Lubin, J. H., Stewart, P. A., Hayes, R. B., Hoover, R. N., & Hauptmann, M. (2013). Mortality from solid tumors among workers in formaldehyde industries: an update of the NCI cohort. *American Journal of Industrial Medicine*, *56*(9), 1015-1026.

Benn, T., & Osborne, K. (1998). Mortality of United Kingdom acrylonitrile workers--an extended and updated study. *Scandanavian Journal of Work, Environment & Health, 24*(Suppl 2), 17-24.

Blair, A., Stewart, P. A., Zaebst, D. D., Pottern, L. M., Zey, J. N., Bloom, T. F., . . . & Lubin, J. (1998). Mortality of industrial workers exposed to acrylonitrile. *Scandanavian Journal of Work, Environment and Health, 24*(Suppl 2), 25-41.

Blair, A., Zheng, T., Linos, A., Stewart, P. A., Zhang, Y. W., & Cantor, K. P. (2001). Occupation and leukemia: a population-based case-control study in Iowa and Minnesota. *American Journal of Industrial Medicine, 40*(1), 3-14.

Bloemen, L. J., Youk, A., Bradley, T. D., Bodner, K. M., & Marsh, G. (2004). Lymphohaematopoietic cancer risk among chemical workers exposed to benzene. *Occupational & Environmental Medicine, 61*(3), 270-274.

Boice, J. D., Jr., Marano, D. E., Fryzek, J. P., Sadler, C. J., & McLaughlin, J. K. (1999). Mortality among aircraft manufacturing workers. *Occupational & Environmental Medicine, 56*(9), 581-597.

Bruning, T., Pesch, B., Wiesenhutter, B., Rabstein, S., Lammert, M., Baumuller, A., & Bolt, H. M. (2003). Renal cell cancer risk and occupational exposure to trichloroethylene: results of a consecutive case-control study in Arnsberg, Germany. *American Journal of Industrial Medicine, 43*(3), 274-285.

Charbotel, B., Fevotte, J., Hours, M., Martin, J. L., & Bergeret, A. (2006). Case-control study on renal cell cancer and occupational exposure to trichloroethylene. Part II: Epidemiological aspects. *Annals of Occupational Hygiene, 50*(8), 777-787.

Checkoway, H., Dell, L. D., Boffetta, P., Gallagher, A. E., Crawford, L., Lees, P. S., & Mundt, K. A. (2015). Formaldehyde exposure and mortality risks from acute myeloid leukemia and other lymphohematopoietic malignancies in the US National Cancer Institute Cohort Study of Workers in Formaldehyde Industries. [Research Support, Non-U.S. Gov't]. *Journal of Occupational & Environmental Medicine, 57*(7), 785-794.

Chen, J. L., Walrath, J., O'Berg, M. T., Burke, C. A., & Pell, S. (1987). Cancer incidence and mortality among workers exposed to acrylonitrile. *American Journal of Industrial Medicine, 11*(2), 157-163.

Cheng, H., Sathiakumar, N., Graff, J., Matthews, R., & Delzell, E. (2007). 1,3-Butadiene and leukemia among synthetic rubber industry workers: exposure-response relationships. *Chemico-Biological Interactions, 166*(1-3), 15-24.

Christensen, K. Y., Vizcaya, D., Richardson, H., Lavoue, J., Aronson, K., & Siemiatycki, J. (2013). Risk of selected cancers due to occupational exposure to chlorinated solvents in a case-control study in Montreal. [Research Support, Non-U.S. Gov't]. *Journal of Occupational & Environmental Medicine, 55*(2), 198-208. doi: 10.1097/JOM.0b013e3182728eab

Christensen, M. S., Vestergaard, J. M., d'Amore, F., Gorlov, J. S., Toft, G., Ramlau-Hansen, C. H., . . . & Kolstad, H. A. (2018). Styrene exposure and risk of lymphohematopoietic malignancies in 73,036 reinforced plastics workers. *Epidemiology, 29*(3), 342-351. doi: 10.1097/EDE.0000000000000819

Cocco, P., T'Mannetje, A., Fadda, D., Melis, M., Becker, N., de, S. S., . . . & Boffetta, P. (2010). Occupational exposure to solvents and risk of lymphoma subtypes: results from the Epilymph case-control study. *Occupational & Environmental Medicine., 67*(5), 341-347. doi: 67/5/341 [pii];10.1136/oem.2009.046839 [doi]

Coggon, D., Harris, E. C., Poole, J., & Palmer, K. T. (2004). Mortality of workers exposed to ethylene oxide: extended follow up of a British cohort. *Occupational & Environmental Medicine, 61*(4), 358-362.

Coggon, D., Ntani, G., Harris, E. C., & Palmer, K. T. (2014). Upper airway cancer, myeloid leukemia, and other cancers in a cohort of British chemical workers exposed to formaldehyde. *American Journal of Epidemiology., 179*(11), 1301-1311. doi: kwu049 [pii];10.1093/aje/kwu049 [doi]

Coggon, D., Ntani, G., Harris, E. C., & Palmer, K. T. (2015). Risk of cancer in workers exposed to styrene at eight British companies making glass-reinforced plastics. [Research Support, Non-U.S. Gov't]. *Occup Environ Med, 72*(3), 165-170. doi: 10.1136/oemed-2014-102382

Collins, J. J., Bodner, K. M., & Bus, J. S. (2013). Cancer mortality of workers exposed to styrene in the U.S. Reinforced plastics and composite industry. *Epidemiology, 24*(2), 195-203. doi: 10.1097/EDE.0b013e318281a30f

Collins, J. J., Ireland, B., Buckley, C. F., & Shepperly, D. (2003). Lymphohaematopoeitic cancer mortality among workers with benzene exposure. *Occupational & Environmental Medicine, 60*(9), 676-679.

Collins, J. J., Page, L. C., Caporossi, J. C., Utidjian, H. M., & Lucas, L. J. (1989). Mortality patterns among employees exposed to acrylonitrile. *Journal of Occupational Medicine, 31*(4), 368-371.

Crump, K. S. (1994). Risk of benzene-induced leukemia: a sensitivity analysis of the pliofilm cohort with additional follow-up and new exposure estimates. *Journal of Toxicology and Environmental Health, 42*(2), 219-242.

Crump, K. S., & Allen, B. C. (1984). *Quantitative Estimates of Risk of Leukemia from Occupational Exposure to Benzene. Prepared for the Occupational Safety and Heal Administration by Science Research Systems, Inc.*  Unpublished. As cited in U.S. Environmental Protection Agency, 2000.

Delzell, E.,

Delzell, E., Macaluso, M., Sathiakumar, N., & Matthews, R. (2001). Leukemia and exposure to 1,3-butadiene, styrene and dimethyldithiocarbamate among workers in the synthetic rubber industry. *Chemico-Biological Interactions, 135-136*, 515-534.

Delzell, E., Sathiakumar, N., Graff, J., Macaluso, M., Maldonado, G., & Matthews, R. (2006). An updated study of mortality among North American synthetic rubber industry workers. *Research Report (Health Effects Institute),* (132), 1-63.

Delzell, E., Sathiakumar, N., Macaluso, M., Hovinga, M., Larson, R., Barbone, F., . . . & Muir, D. C. (1996). A folow-up study of synthetic rubber workers. Toxicology, 113(1-3), 182-189

Divine, B. J., & Hartman, C. M. (2001). A cohort mortality study among workers at a 1,3 butadiene facility. *Chemico-Biological Interactions, 135-136*, 535-553.

EPA. (1987). Acrylonitrile. CASRN 107-13-1. Washington, DC: US Environmental Protection Agency. Accessed 2018. <https://cfpub.epa.gov/ncea/iris2/chemicalLanding.cfm?substance_nmbr=206>

EPA. (1998). Carcinogenic effects of benzene: an update. Integrated Risk Information System (IRIS). EPA/600/P-97/001F. Washington, DC: National Center for Environmental Health, Office of Research and Development.

EPA. (2002). Health Assessment of 1,3-Butadiene. EPA/600/P-98/001F. Washington, DC: National Center for Environmental Assessment. Office of Research and Development. October 2002.

EPA. (2010). Toxicological Review of Formaldehyde - Inhalation Assessment (CAS No. 50-00-0). (External Review Draft 2010). (Vol. Volume III). Washington, DC; U.S. Environmental Protection Agency. June 2010. www.epa.gov/iris

EPA. (2011a). IRIS Toxicological Review of Acrylonitrile (External Review Draft). Washington, DC: US Environmental Protection Agency. <https://cfpub.epa.gov/ncea/iris_drafts/recordisplay.cfm?deid=198583>

EPA. (2011b). Toxicological review of Dichloromethane (methylene chloride). CAS No. 75-09-2. Washington, DC. U.S. Environmental Protection Agency. November 2011. ww.epa.gov/iris

EPA. (2011c). Toxicological Review of Trichloroethylene (CAS No. 70-01-6). In support of summary information on the Integrated Risk Information System (IRIS). (EPA/635/R-09/011F). Washington, DC: U.S. Environmental Protection Agency. September 2011. <http://www.epa.gov/iris>.

EPA. (2016). Evaluation of the Inhalation Carcinogenicity of Ethylene Oxide (CASRN 75-21-8) In Support of Summary Information on the Integrated Risk Information System (IRIS). U.S. Environmental Protection Agency, Washington, DC, EPA/635/R-16/350Fa, 2016.

Gibbs, G. W., Amsel, J., & Soden, K. (1996). A cohort mortality study of cellulose triacetate-fiber workers exposed to methylene chloride. *Journal of Occupational & Environmental Medicine, 38*(7), 693-697.

Glass, D. C., Adams, G. G., Manuell, R. W., & Bisby, J. A. (2000). Retrospective exposure assessment for benzene in the Australian petroleum industry. *Annals of Occupational Hygiene, 44*(4), 301-320.

Glass, D. C., Gray, C. N., Jolley, D. J., Gibbons, C., Sim, M. R., Fritschi, L., . . . & Manuell, R. (2003). Leukemia risk associated with low-level benzene exposure. *Epidemiology, 14*(5), 569-577.

Glass, D. C., Schnatter, A. R., Tang, G., Irons, R. D., & Rushton, L. (2014). Risk of myeloproliferative disease and chronic myeloid leukaemia following exposure to low-level benzene in a nested case-control study of petroleum workers. *Occupational & Environmental Medicine., 71*(4), 266-274. doi: oemed-2013-101664 [pii];10.1136/oemed-2013-101664 [doi]

Gold, L. S., Stewart, P. A., Milliken, K., Purdue, M., Severson, R., Seixas, N., . . . & De Roos, A. J. (2011). The relationship between multiple myeloma and occupational exposure to six chlorinated solvents. *Occupational & Environmental Medicine, 68*, 391-399.

Graff, J. J., Sathiakumar, N., Macaluso, M., Maldonado, G., Matthews, R., & Delzell, E. (2005). Chemical exposures in the synthetic rubber industry and lymphohematopoietic cancer mortality. *Journal of Occupational & Environmental Medicine, 47*(9), 916-932.

Greenberg, H. L., Ott, M. G., & Shore, R. E. (1990). Men assigned to ethylene oxide production or other ethylene oxide related chemical manufacturing: a mortality study. *British Journal of Industrial Medicine, 47*(4), 221-230.

Guenel, P., Imbernon, E., Chevalier, A., Crinquand-Calastreng, A., & Goldberg, M. (2002). Leukemia in relation to occupational exposures to benzene and other agents: a case-control study nested in a cohort of gas and electric utility workers. *American Journal of Industrial Medicine, 42*(2), 87-97.

Hauptmann, M., Stewart, P. A., Lubin, J. H., Beane Freeman, L. E., Hornung, R. W., Herrick, R. F., . . . & Hayes, R. B. (2009). Mortality from lymphohematopoietic malignancies and brain cancer among embalmers exposed to formaldehyde. *Journal of the National Cancer Institute., 101*(24), 1696-1708. doi: djp416 [pii];10.1093/jnci/djp416 [doi]

Hayes, R. B., Yin, S. N., Dosemeci, M., Li, G. L., Wacholder, S., Chow, W. H., . . . & Linet, M. S. (1996). Mortality among benzene-exposed workers in China. *Environmental Health Perspectives, 104 Suppl 6*, 1349-1352.

Hayes, R. B., Yin, S. N., Dosemeci, M., Li, G. L., Wacholder, S., Travis, L. B., . . . & Linet, M. S. (1997). Benzene and the dose-related incidence of hematologic neoplasms in China. Chinese Academy of Preventive Medicine--National Cancer Institute Benzene Study Group. *Journal of the National Cancer Institute, 89*(14), 1065-1071.

Hearne, F. T., & Pifer, J. W. (1999). Mortality study of two overlapping cohorts of photographic film base manufacturing employees exposed to methylene chloride. *Journal of Occupational & Environmental Medicine, 41*(12), 1154-1169.

Hildesheim, A., Dosemeci, M., Chan, C. C., Chen, C. J., Cheng, Y. J., Hsu, M. M., . . . & Yang, C. S. (2001). Occupational exposure to wood, formaldehyde, and solvents and risk of nasopharyngeal carcinoma. *Cancer Epidemiology, Biomarkers & Prevention, 10*(11), 1145-1153.

Hogstedt, C., Aringer, L., & Gustavsson, A. (1986). Epidemiologic support for ethylene oxide as a cancer-causing agent. *Journal of the American Medical Association, 255*(12), 1575-1578.

IARC. (1982). *IARC Monographs on the Evaluation of the Carcinogenic Risk of Chemicals to Humans: Some industrial chemicals and dyestuffs.* Volume 29. Lyon, France. International Agency for Research on Cancer (IARC): World Health Organization.

IARC. (2006). *IARC Monographs on the Evaluation of the Carcinogenic Risks to Humans* *Formaldehyde, 2-Butoxyethanol and 1-tert-Butoxypropan-2-ol*. *Volume 88.* Lyon, France: World Health Organization; International Agency for Research on Cancer.

IARC. (2008). *IARC Monographs on the evaluation of Carcinogenic Risks to Humans. Volume 97. 1,3-Butadiene, Ethylene Oxide and Vinyl Halides (Vinyl Fluoride, Vinyl Chloride and Vinyl Bromide).* Lyon, France: World Health Organization (WHO). International agency for Research on Cancer (IARC).

IARC. (2012). *IARC Monographs on the Evaluation of Carcinogenic Risks to Humans. Volume 100. A Review of Human Carcinogens Part F: Chemical Agents and Related Occupations*. Lyon, France: International Agency for Research on Cancer (IARC; World Health Organization (WHO).

IARC. (2014). *IARC Monographs on the evaluation of Carcinogenic Risks. Volume 106. Trichloroethylene, Tetrachloroethylene, and some other chlorinated agents.* *Volume 106.* Lyon, France. World Health Organization; International Agency for Research on Cancer (IARC).

IARC. (2017). *IARC Monographs on the evaluation of carcinogenic risks to humans. Some chemicals used as solvents and in polymer manufacture.* *Volume 110*. Lyon France, International Agency for Research on Cancer (IARC). World Health Organization.

Jarup, L., Pershagen, G., & Wall, S. (1989). Cumulative arsenic exposure and lung cancer in smelter workers: a dose-response study. [Research Support, Non-U.S. Gov't]. *American Journal of Industrial Medicine, 15*(1), 31-41.

Kogevinas, M., Ferro, G., Andersen, A., Bellander, T., Biocca, M., Coggon, D., . . . . & Lundberg, I. (1994). Cancer mortality in a historical cohort study of workers exposed to styrene. *Scandanavian Journal of Work, Environment & Health, 20*(4), 251-261. doi: 1400 [pii]

Kogevinas, M., Sim, M., Parent, M. E., Calaf, G. M., PVodicka, J., Jensen, A. A., . . . & International Agency for Research on Cancer Monograph Working Group. (2018). Carcinogenicity of quinoline, styrene, and styrene-7,8-oxide. *The Lancet*.

Kolstad, H. A., Sonderskov, J., & Burstyn, I. (2005). Company-level, semi-quantitative assessment of occupational styrene exposure when individual data are not available. [Research Support, Non-U.S. Gov't]. *Annals of Occupational Hygiene, 49*(2), 155-165. doi: 10.1093/annhyg/meh088

Laforest, L., Luce, D., Goldberg, P., Begin, D., Gerin, M., Demers, P. A., . . . & Leclerc, A. (2000). Laryngeal and hypopharyngeal cancers and occupational exposure to formaldehyde and various dusts: a case-control study in France. *Occupational & Environmental Medicine, 57*(11), 767-773.

Lanes, S. F., Rothman, K. J., Dreyer, N. A., & Soden, K. J. (1993). Mortality update of cellulose fiber production workers. *Scandanavian Journal of Work, Environment & Health, 19*(6), 426-428.

Lewis, R. J., Schnatter, A. R., Drummond, I., Murray, N., Thompson, F. S., Katz, A. M., . . . & Theriault, G. (2003). Mortality and cancer morbidity in a cohort of Canadian petroleum workers. *Occupational & Environmental Medicine, 60*(12), 918-928.

Linet, M. S., Yin, S. N., Gilbert, E. S., Dores, G. M., Hayes, R. B., Vermeulen, R., . . . & Rothman, N. (2015). A retrospective cohort study of cause-specific mortality and incidence of hematopoietic malignancies in chinese benzene-exposed workers. *International Journal of Cancer, 137*(9), 2184-2197. doi: 10.1002/ijc.29591 [doi]

Lipworth, L., Sonderman, J. S., Mumma, M. T., Tarone, R. E., Marano, D. E., Boice, J. D., Jr., & McLaughlin, J. K. (2011). Cancer mortality among aircraft manufacturing workers: an extended follow-up. *Journal of Occupational & Environmental Medicine, 53*(9), 992-1007. doi: 10.1097/JOM.0b013e31822e0940 [doi]

Loomis, D., Guyton, K. Z., Grosse, Y., El Ghissassi, F., Bouvard, V., Benbrahim-Tallaa, L., . . . & International Agency for Research on Cancer Monograph Working Group. (2017). Carcinogenicity of benzene. *Lancet Oncology*. doi: 10.1016/S1470-2045(17)30832-X

Luce, D., Gerin, M., Leclerc, A., Morcet, J. F., Brugere, J., & Goldberg, M. (1993). Sinonasal cancer and occupational exposure to formaldehyde and other substances. *International Journal of Cancer, 53*(2), 224-231.

Macaluso, M., Larson, R., Delzell, E., Sathiakumar, N., Hovinga, M., Julian, J., . . . & Cole, P. (1996). Leukemia and cumulative exposure to butadiene, styrene and benzene among workers in the synthetic rubber industry. *Toxicology, 113*(1-3), 190-202.

Marsh, G. M., Gula, M. J., Youk, A. O., & Schall, L. C. (1999). Mortality among chemical plant workers exposed to acrylonitrile and other substances. *American Journal of Industrial Medicine, 36*(4), 423-436.

Marsh, G. M., Morfeld, P., Zimmerman, S. D., Liu, Y., & Balmert, L. C. (2016). An updated re-analysis of the mortality risk from nasopharyngeal cancer in the National Cancer Institute formaldehyde worker cohort study. *Journal of Occupational Medicine & Toxicology, 11*, 8. doi: 10.1186/s12995-016-0097-6

Marsh, G. M., Youk, A. O., & Collins, J. J. (2001). Re-evaluation of lung cancer risk in the acrylonitrile cohort study of the National Cancer Institute and the National Institute for Occupational Safety and Health. *Scandanavian Journal of Work, Environment & Health, 27*(1), 5-13.

Marsh, G. M., Youk, A. O., Stone, R. A., Buchanich, J. M., Gula, M. J., Smith, T. J., & Quinn, M. M. (2001). Historical cohort study of US man-made vitreous fiber production workers: I. 1992 fiberglass cohort follow-up: initial findings. [Comparative Study Research Support, Non-U.S. Gov't]. *Journal of Occupational & Environmental Medicine, 43*(9), 741-756.

Matanoski, G., Elliott, E., Tao, X., Francis, M., Correa-Villasenor, A., & Santos-Burgoa, C. (1997). Lymphohematopoietic cancers and butadiene and styrene exposure in synthetic rubber manufacture. *Annals of the New York Academy of Science, 837*, 157-169.

Meyers, A. R., Pinkerton, L. E., & Hein, M. J. (2013). Cohort mortality study of garment industry workers exposed to formaldehyde: Update and internal comparisons. *American Journal of Industrial Medicine, 59*(9), 1027-1039. doi: 10.1002/ajim.22199 [doi]

Moore, L. E., Boffetta, P., Karami, S., Brennan, P., Stewart, P. S., Hung, R., . . . & Rothman, N. (2010). Occupational trichloroethylene exposure and renal carcinoma risk: evidence of genetic susceptibility by reductive metabolism gene variants. [Research Support, N.I.H., Intramural]. *Cancer Research, 70*(16), 6527-6536. doi: 10.1158/0008-5472.CAN-09-4167

Morgan, R. W., Claxton, K. W., Divine, B. J., Kaplan, S. D., & Harris, V. B. (1981). Mortality among ethylene oxide workers. *Journal of Occupational Medicine, 23*(11), 767-770.

Morgan, R. W., Kelsh, M. A., Zhao, K., & Heringer, S. (1998). Mortality of aerospace workers exposed to trichloroethylene. *Epidemiology, 9*(4), 424-431.

Nissen, M. S., Stokholm, Z. A., Christensen, M. S., Schlunssen, V., Vestergaard, J. M., Iversen, I. B., & Kolstad, H. A. (2018). Sinonasal adenocarcinoma following styrene exposure in the reinforced plastics industry. *Occupational & Environmental Medicine, 75*(6), 412-414. doi: 10.1136/oemed-2017-104974

O'Berg, M. T. (1980). Epidemiologic study of workers exposed to acrylonitrile. *Journal of Occupational Medicine, 22*(4), 245-252.

O'Berg, M. T., Chen, J. L., Burke, C. A., Walrath, J., & Pell, S. (1985). Epidemiologic study of workers exposed to acrylonitrile: an update. *Journal of Occupational Medicine, 27*(11), 835-840.

Ott, M. G., Skory, L. K., Holder, B. B., Bronson, J. M., & Williams, P. R. (1983). Health evaluation of employees occupationally exposed to methylene chloride: Mortality. *Scandanavian Journal of Work, Environment & Health, 9*(suppl 1), 8-16.

Partanen, T., Kauppinen, T., Luukkonen, R., Hakulinen, T., & Pukkala, E. (1993). Malignant lymphomas and leukemias, and exposures in the wood industry: an industry-based case-referent study. *International Archives of Occupational & Environmental Health, 64*(8), 593-596.

Paustenbach, D. J., Bass, R. D., & Price, P. (1993). Benzene toxicity and risk assessment, 1972-1992: implications for future regulation. *Environmental Health Perspectives, 101 Suppl 6*, 177-200.

Pesch, B., Haerting, J., Ranft, U., Klimpel, A., Oelschlagel, B., & Schill, W. (2000). Occupational risk factors for renal cell carcinoma: agent-specific results from a case-control study in Germany. MURC Study Group: Multicenter urothelial and renal cancer study. *International Journal of Epidemiology, 29*(6), 1014-1024.

Pira, E., Romano, C., Verga, F., & La, V. C. (2014). Mortality from lymphohematopoietic neoplasms and other causes in a cohort of laminated plastic workers exposed to formaldehyde. *Cancer Causes & Control, 25*(10), 1343-1349. doi: 10.1007/s10552-014-0440-0 [doi]

Purdue, M. P., Bakke, B., Stewart, P., De Roos, A. J., Schenk, M., Lynch, C. F., . . . & Colt, J. S. (2011). A case-control study of occupational exposure to trichloroethylene and Non-Hodgkin Lymphoma. *Environmental Health Perspectives, 119*(2), 232-238.

Raaschou-Nielsen, O., Hansen, J., McLaughlin, J. K., Kolstad, H., Christensen, J. M., Tarone, R. E., & Olsen, J. H. (2003). Cancer risk among workers at Danish companies using trichloroethylene: a cohort study. *American Journal of Epidemiology, 158*(12), 1182-1192.

Raaschou-Nielsen, O., Hertel, O., Thomsen, B. L., & Olsen, J. H. (2001). Air pollution from traffic at the residence of children with cancer. *American Journal of Epidemiology, 153*(5), 433-443.

Radican, L., Blair, A., Stewart, P., & Wartenberg, D. (2008). Mortality of aircraft maintenance workers exposed to trichloroethylene and other hydrocarbons and chemicals: extended follow-up. *Journal of Occupational & Environmental Medicine, 50*(11), 1306-1319.

Rinsky, R. A., Hornung, R. W., Silver, S. R., & Tseng, C. Y. (2002). Benzene exposure and hematopoietic mortality: A long-term epidemiologic risk assessment. *American Journal of Industrial Medicine, 42*(6), 474-480.

Rinsky, R. A., Smith, A. B., Hornung, R., Filloon, T. G., Young, R. J., Okun, A. H., & Landrigan, P. J. (1987). Benzene and leukemia: an epidemiologic risk assessment. *New England Journal of Medicine, 316*(17), 1044-1050.

Rinsky, R. A., Young, R. J., & Smith, A. B. (1981). Leukemia in benzene workers. *American Journal of Industrial Medicine, 2*(3), 217-245.

Rushton, L., & Romaniuk, H. (1997). A case-control study to investigate the risk of leukaemia associated with exposure to benzene in petroleum marketing and distribution workers in the United Kingdom. *Occupational & Environmental Medicine, 54*(3), 152-166.

Saberi Hosnijeh, F., Christopher, Y., Peeters, P., Romieu, I., Xun, W., Riboli, E., . . . & Vermeulen, R. (2013). Occupation and risk of lymphoid and myeloid leukaemia in the European Prospective Investigation into Cancer and Nutrition (EPIC). *Occupational & Environmental Medicine., 70*(7), 464-470. doi: oemed-2012-101135 [pii];10.1136/oemed-2012-101135 [doi]

Sathiakumar, N., Delzell, E., Hovinga, M., Macaluso, M., Julian, J. A., Larson, R., . . . & Muir, D. C. (1998). Mortality from cancer and other causes of death among synthetic rubber workers. *Occupational & Environmental Medicine, 55*(4), 230-235.

Schnatter, A. R., Armstrong, T. W., Nicolich, M. J., Thompson, F. S., Katz, A. M., Huebner, W. W., & Pearlman, E. D. (1996). Lymphohaematopoietic malignancies and quantitative estimates of exposure to benzene in Canadian petroleum distribution workers. *Occupational & Environmental Medicine, 53*(11), 773-781.

Schnatter, A. R., Glass, D. C., Tang, G., Irons, R. D., & Rushton, L. (2012). Myelodysplastic Syndrome and Benzene Exposure Among Petroleum Workers: An International Pooled Analysis. *Journal of the National Cancer Institute, 104*(22), 1724-1737. doi: djs411 [pii];10.1093/jnci/djs411 [doi]

Seidler, A., Mohner, M., Berger, J., Mester, B., Deeg, E., Elsner, G., . . . & Becker, N. (2007). Solvent exposure and malignant lymphoma: a population-based case-control study in Germany. *Journal of Occupational Medicine & Toxicology, 2*(1), 2.

Stayner, L., Steenland, K., Greife, A., Hornung, R., Hayes, R. B., Nowlin, S., . . . & Halperin, W. (1993). Exposure-response analysis of cancer mortality in a cohort of workers exposed to ethylene oxide. *American Journal of Epidemiology, 138*(10), 787-798.

Steenland, K., Stayner, L., & Deddens, J. (2004). Mortality analyses in a cohort of 18 235 ethylene oxide exposed workers: follow up extended from 1987 to 1998. *Occupational & Environmental Medicine, 61*(1), 2-7.

Steenland, K., Whelan, E., Deddens, J., Stayner, L., & Ward, E. (2003). Ethylene oxide and breast cancer incidence in a cohort study of 7576 women (United States). *Cancer Cause &s Control, 14*(6), 531-539.

Stenehjem, J. S., Kjaerheim, K., Bratveit, M., Samuelsen, S. O., Barone-Adesi, F., Rothman, N., . . . Grimsrud, T. K. (2015). Benzene exposure and risk of lymphohaematopoietic cancers in 25 000 offshore oil industry workers. *British Journal of Cancer, 112*(9), 1603-1612. doi: bjc2015108 [pii];10.1038/bjc.2015.108 [doi]

Stewart, P. A., Blair, A., Cubit, D., Bales, R., Kaplan, S. A., Ward, J., . . . & Walrath, J. (1986). Estimating historical exposures to formaldehyde in a retrospective mortality study. *Applied Industrial Hygiene, 1*(1), 34-41.

Stewart, P. A., Lee, J. S., Marano, D. E., Spirtas, R., Forbes, C. D., & Blair, A. (1991). Retrospective cohort mortality study of workers at an aircraft maintenance facility. II. Exposures and their assessment. *British Journal of Industrial Medicine*, *48*(8), 531-537.

Stewart, P. A., Zaebst, D. D., Zey, J. N., Herrick, R., Dosemeci, M., Hornung, R., . . . & Blair, A. (1998). Exposure assessment for a study of workers exposed to acrylonitrile. *Scandanavian Journal of Work, Environment & Health, 24*(Suppl 2), 42-53.

Swaen, G. M., Bloemen, L. J., Twisk, J., Scheffers, T., Slangen, J. J., Collins, J. J., & ten Berge, W. F. (2004). Mortality update of workers exposed to acrylonitrile in The Netherlands. *Journal of Occupational & Environmental Medicine, 46*(7), 691-698.

Swaen, G. M., Bloemen, L. J., Twisk, J., Scheffers, T., Slangen, J. J., Collins, J. J., . . . & Sturmans, F. (1998). Mortality update of workers exposed to acrylonitrile in the Netherlands. *Scandanavian Journal of Work,Environment & Health, 24*(Suppl 2), 10-16.

Swaen, G. M., Bloemen, L. J., Twisk, J., Scheffers, T., Slangen, J. J., & Sturmans, F. (1992). Mortality of workers exposed to acrylonitrile. *Journal of Occupational Medicine, 34*(8), 801-809.

Swaen, G. M., Burns, C. J., Teta, J. M., Bodner, K. M., Keenan, D., & Bodnar, C. M. (2009). Mortality study update of ethylene oxide workers in chemical manufacturing: a 15 year update. *Journal of Occupational & Environmental Medicine, 51*(6), 714-723.

Symons, J. M., Kreckmann, K. H., Sakr, C. J., Kaplan, A. M., & Leonard, R. C. (2008). Mortality among workers exposed to acrylonitrile in fiber production: an update. [Comparative Study]. *Journal of Occupational & Environmetnal Medicine, 50*(5), 550-560. doi: 10.1097/JOM.0b013e318162f640

Talibov, M., Lehtinen-Jacks, S., Martinsen, J. I., Kjaerheim, K., Lynge, E., Sparen, P., . . . & Pukkala, E. (2014). Occupational exposure to solvents and acute myeloid leukemia: a population-based, case-control study in four Nordic countries. *Scandanavian Journal of Work, Environment & Health, 40*(5), 511-517. doi: 3436 [pii];10.5271/sjweh.3436 [doi]

Tarvainen, L., Kyyronen, P., Kauppinen, T., & Pukkala, E. (2008). Cancer of the mouth and pharynx, occupation and exposure to chemical agents in Finland [in 1971-95]. *International Journal of Cancer, 123*(3), 653-659. doi: 10.1002/ijc.23286 [doi]

Tomenson, J. A. (2011). Update of a cohort mortality study of workers exposed to methylene chloride employed at a plant producing cellulose triacetate film base. *International Archives of Occupational & Environmental Health, 84*(8), 889-897. doi: 10.1007/s00420-011-0621-z [doi]

Tomenson, J. A., Bonner, S. M., Heijne, C. G., Farrar, D. G., & Cummings, T. F. (1997). Mortality of workers exposed to methylene chloride employed at a plant producing cellulose triacetate film base [see comments]. *Occupational & Environmental Medicine, 54*(7), 470-476.

Valdez-Flores, C., Sielken, R. L., Jr., & Teta, M. J. (2010). Quantitative cancer risk assessment based on NIOSH and UCC epidemiological data for workers exposed to ethylene oxide. [Research Support, Non-U.S. Gov't]. *Regulatory Toxicology & Pharmacology, 56*(3), 312-320. doi: 10.1016/j.yrtph.2009.10.001

Vamvakas, S., Bruning, T., Thomasson, B., Lammert, M., Baumuller, A., Bolt, H. M., . . . & Ulm, K. (1998). Renal cell cancer correlated with occupational exposure to trichloroethene [see comments]. *Journal of Cancer Research and Clinical Oncology, 124*(7), 374-382.

Vaughan, T. L., Stewart, P. A., Teschke, K., Lynch, C. F., Swanson, G. M., Lyon, J. L., & Berwick, M. (2000). Occupational exposure to formaldehyde and wood dust and nasopharyngeal carcinoma. *Occupational and Environmental Medicine, 57*(6), 376-384.

Wang, R., Zhang, Y., Lan, Q., Holford, T. R., Leaderer, B., Hoar Zahm, S., . . . & Zheng, T. Z. (2009). Occupational Exposure to Solvents and Risk of Non-Hodgkin Lymphoma in Connecticut Women. *American Journal of Epidemiology, 169*(2), 176-185.

West, R. R., Stafford, D. A., Farrow, A., & Jacobs, A. (1995). Occupational and environmental exposures and myelodysplasia: a case-control study. *Leukemia Research, 19*(2), 127-139.

Wong, O. (1987). An industry wide mortality study of chemical workers occupationally exposed to benzene. II. Dose response analyses. *British Journal of Industrial Medicine, 44*(6), 382-395.

Wong, O. (1995). Risk of acute myeloid leukaemia and multiple myeloma in workers exposed to benzene. *Occupational & Environmental Medicine, 52*(6), 380-384.

Wong, O., Harris, F., & Smith, T. J. (1993). Health effects of gasoline exposure. II. Mortality patterns of distribution workers in the United States. *Environmental Health Perspectives, 101*(Suppl 6), 63-76.

Wood, S. M., Buffler, P. A., Burau, K., & Krivanek, N. (1998). Mortality and morbidity of workers exposed to acrylonitrile in fiber production. *Scandanavian Journal of Work, Environment & Health, 24*(Suppl 2), 54-62.

Yin, S. N., Hayes, R. B., Linet, M. S., Li, G. L., Dosemeci, M., Travis, L. B., . . . & Blot, W. J. (1996). A cohort study of cancer among benzene-exposed workers in China: overall results. *American Journal of Industrial Medicine, 29*(3), 227-235.

Youk, A. O., Marsh, G. M., Stone, R. A., Buchanich, J. M., & Smith, T. J. (2001). Historical cohort study of US man-made vitreous fiber production workers: III. Analysis of exposure-weighted measures of respirable fibers and formaldehyde in the nested case-control study of respiratory system cancer. *Journal of Occupational & Environmental Medicine, 43*(9), 767-778.

Zhao, Y., Krishnadasan, A., Kennedy, N., Morgenstern, H., & Ritz, B. (2005). Estimated effects of solvents and mineral oils on cancer incidence and mortality in a cohort of aerospace workers. *American Journal of Industrial Medicine, 48*(4), 249-258.
